# Revealing hidden diversity in gonostomatid ciliates (Ciliophora, Hypotrichia): integrative evidence supports two new species, a new genus, and Wallackiidae fam. nov.

**DOI:** 10.1186/s12866-026-05385-x

**Published:** 2026-07-11

**Authors:** Atef Omar, Jae-Ho Jung

**Affiliations:** 1 EcoBio Research Center, Kangwon National University, Gangneung, 25457 South Korea; 2 Department of Biological Sciences, Kangwon National University, Gangneung, 25457 South Korea

**Keywords:** Morphology, Morphogenesis, *Metagonostomoides elongatus* gen. nov., sp. nov., Multigene phylogeny, *Wallackia koreana* sp. nov.

## Abstract

**Background:**

Gonostomatidae are among the most common terrestrial hypotrichs and are readily recognized by a distinctive oral apparatus (knee-shaped adoral zone of membranelles; monokinetidal undulating membranes with a widely spaced paroral and densely spaced endoral). A further key diagnostic trait is morphogenesis, in which the proter and opisthe share primary frontal-ventral-transverse (FVT) primordia that later split into secondary primordia. Despite their apparent diversity in soils, comparatively few gonostomatid species have been described, and most lack complete morphogenetic and/or molecular documentation. This insufficient documentation obscures relationships and contributes to taxonomic instability, highlighting the need for integrative taxonomy combining morphology, detailed morphogenesis, and multigene phylogenetics.

**Results:**

From Korean soils we discovered two novel hypotrichs with a gonostomatid oral apparatus. The first is a new gonostomatid species with five FVT cirral rows and one or two transverse cirri. Its morphogenesis is distinctive within Gonostomatidae: the rightmost cirral row arises predominantly from anlage V with a short posterior contribution from anlage IV (i.e., pseudorow). Phylogenetic analyses show marker-dependent placements: the new species clusters with *Metagonostomum gonostomoidum* in the 18S rDNA tree but with *Paragonostomoides xianicum* in ITS1–5.8S–ITS2, 28S rDNA, and multi-gene datasets, indicating that the 18S rDNA marker lacks sufficient phylogenetic signal unlike the other markers; integrative evidence supports establishment of *Metagonostomoides* gen. nov. The second species, *Wallackia koreana* sp. nov., has a buccal cirral row reduced to two buccal cirri and an adoral zone occupying ~ 62% of body length; it clusters with *W. bujoreani* and *Cladotricha australis*, and the three group with Chaetospiridae in ITS1–5.8S rDNA–ITS2 and multi-gene datasets. A comparative review further shows that *Wallackia* and *Neowallackia* deviate from core gonostomatids by lacking shared primary primordia (proter and opisthe anlagen form independently), supporting Wallackiidae fam. nov. Re-evaluation of the “gonostomatid-type” oral apparatus across hypotrichs restricts it to Gonostomatidae, Chaetospiridae, and Wallackiidae fam. nov.

**Conclusion:**

We document two new soil hypotrich species from Korea, one representing a new gonostomatid genus (*Metagonostomoides* gen. nov.) and the other a new *Wallackia* species (*W. koreana* sp. nov.), and show that their integrative placement helps resolve relationships among hypotrichs with a gonostomatid oral apparatus. The combined morphological, morphogenetic, and multigene phylogenetic evidence supports recognition of Wallackiidae fam. nov. and restricts the gonostomatid-type oral apparatus to Gonostomatidae, Chaetospiridae, and Wallackiidae fam. nov.

**Supplementary Information:**

The online version contains supplementary material available at https://doi.org/10.1186/s12866-026-05385-x.

## Background

Members of the family Gonostomatidae Small & Lynn, 1985 are among the most common ciliate groups in terrestrial habitats [1–5]. Morphologically, they are readily distinguished from other hypotrichs by their conspicuous oral apparatus: the adoral zone of membranelles runs mainly along the left body margin, and its proximal portion bends toward the cell midline to form a characteristic knee-shaped curve; the undulating membranes are monokinetidal, with the paroral composed of widely spaced kinetids and the endoral of densely spaced kinetids [1, 5, 6]. A further diagnostic feature of gonostomatids is their morphogenesis, in which the frontal-ventral-transverse (FVT) anlagen of the proter and opisthe arise as shared primary primordia that subsequently separate to produce the secondary primordia in each daughter cell [1, 5, 7–12]. Previous taxonomic studies on gonostomatids have focused largely on morphology, emphasizing characters such as the presence or absence of long cirral rows, transverse cirri, and caudal cirri. However, many of these studies have overlooked morphogenetic evidence, particularly the origin and development of the FVT anlagen, which can provide critical characters for delimiting taxa and evaluating relationships [4, 5, 13–21].

Despite the high diversity of gonostomatids in soil samples, comparatively few species have been described to date. Most belong to *Gonostomum* Sterki, 1878, and the majority of described species lack morphogenetic and/or molecular data [4, 5]. Even among species for which morphogenesis has been reported, key stages are often missing, especially those that can discriminate taxa at the genus level, such as the origin and development of each FVT anlage [12, 20]. This lack of adequate documentation hampers reliable species delimitation and obscures relationships within the group. To address the likely underestimation of gonostomatid diversity and to resolve the apparent non-monophyly of Gonostomatidae, integrative approaches are required that combine detailed morphological characterization, complete morphogenetic documentation, and multigene phylogenetic analyses. Such approaches would enable more accurate generic assignments and a robust framework for classification.

In the present study, we report two novel hypotrich ciliates with a gonostomatid-type oral apparatus from Korean soils. Using an integrative approach combining morphology, morphogenesis, and multigene phylogenetic analyses, we describe one new genus and species, *Metagonostomoides elongatus* gen. nov., sp. nov., which exhibits a unique ontogenetic pattern representing a previously unknown mode of frontoventral-transverse cirral development within the Gonostomatidae, and one new species, *Wallackia koreana* sp. nov. We further reassess phylogenetic relationships and morphogenetic patterns within gonostomatid-related taxa, leading to the establishment of Wallackiidae fam. nov. Finally, we re-evaluate the distribution of the gonostomatid-type oral apparatus across hypotrichs and discuss its significance for the systematics and evolution of this group.

### ZooBank registration

The ZooBank registration number of present work is: urn:lsid:zoobank.org:pub:7F1213E2-82BE-418F-9F39-69D51B138503.

## Results

### Taxonomy

Class Spirotrichea Bütschli, 1889.

Subclass Hypotricha Stein, 1859.

Family Gonostomatidae Small & Lynn, 1985.

### Genus *Metagonostomoides* gen. nov.

#### ZooBank registration: urn:lsid:zoobank.org:act:408C5732-CD1E-4DB4-AB51-808A50D2C809

#### Diagnosis

Gonostomatidae with five frontal-ventral-transverse cirral rows derived from anlagen I–V. The two rightmost cirral rows are formed within-row at approximately mid-row level, end in second quarter of body, and each usually bears a transverse cirrus between posterior ends of marginal cirral rows; pretransverse ventral cirri absent. The rightmost frontoventral cirral row (frontoterminal) originates from anlage V together with the posteriormost portion of anlage IV. One right and one left marginal cirral row. Three dorsal kineties, each terminating posteriorly in a single caudal cirrus.

#### Type species

*Metagonostomoides elongatus* sp. nov.

#### Etymology

Composite of the generic name *Metagonostomum* Foissner, 2016 and the Greek suffix -*oides* (similar), referring to the similarity with *Metagonostomum*. Masculine gender.

#### Species assignable

Only type species.

### *Metagonostomoides elongatus* sp. nov. (Figs. 1 and 2; Table 1)

**Fig. 1:**
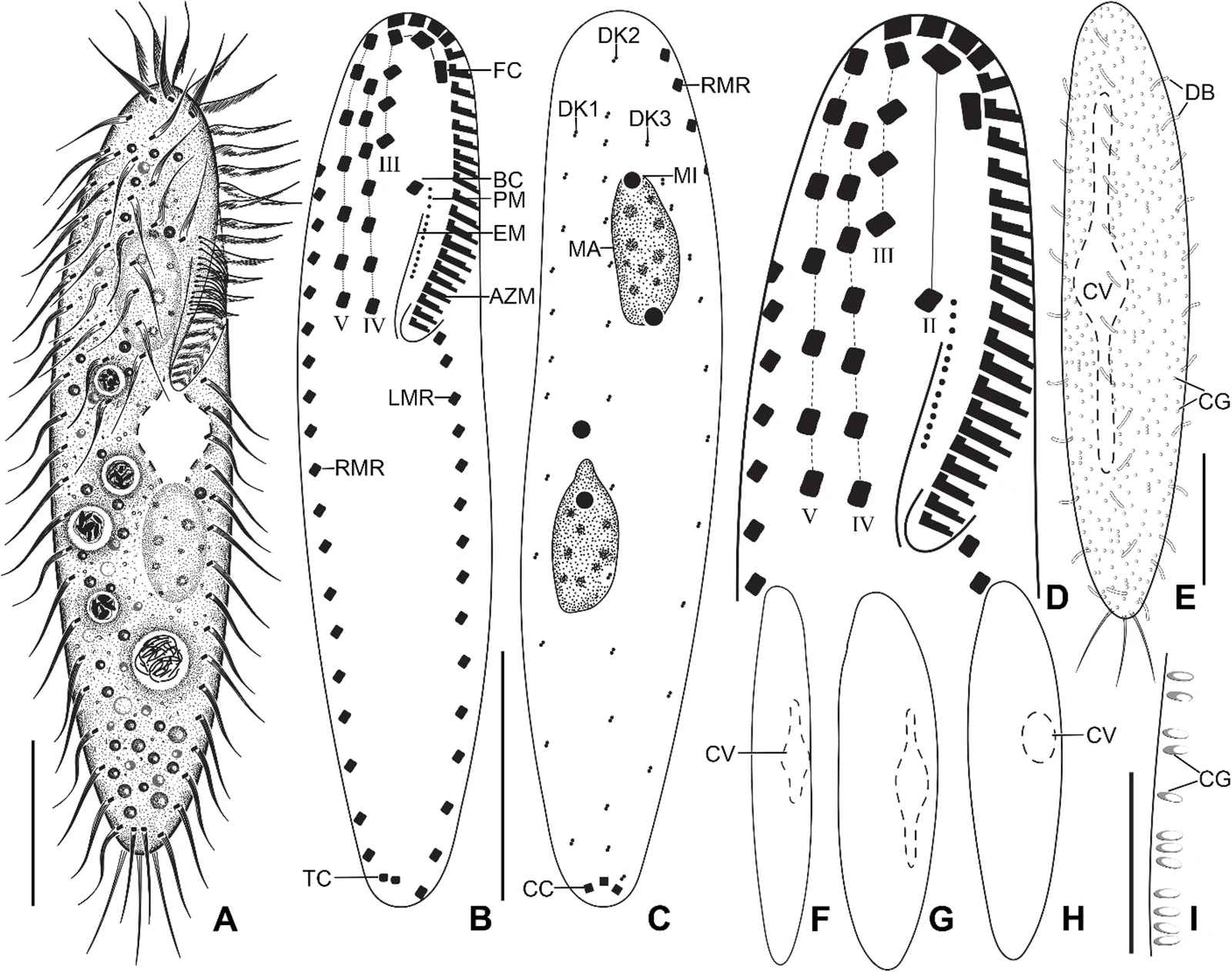
*Metagonostomoides elongatus* sp. nov., from life (**A**, **E**–**I**) and after protargol impregnation (**B**–**D**). **A **Ventral view of a representative specimen, showing, inter alia, the elongated ellipsoidal body shape and the cirral pattern. **B–D **Ventral (**B**, **D**) and dorsal (**C**) view of the holotype specimen, showing the cirral pattern, the undulating membranes in *Gonostomum* pattern, the two transverse cirri, the nuclear apparatus, and the three dorsal kineties. **E**,** I **Dorsal (**E**) view and optical section at cell margin, showing the shape and the random arrangement of cortical granules. **F–H **Ventral views showing different body shapes and contractile vacuole. AZM, adoral zone of membranelles; BC, buccal cirrus; CC, caudal cirri; CG, cortical granules; CV, contractile vacuole; DB, dorsal bristles; DK1–3, dorsal kineties; EM, endoral membrane; FC, frontal cirri; FVC, frontoventral cirri; II–V, frontoventral cirral rows; LMR, left marginal row; MA, macronuclear nodule; MI, micronucleus; PM, paroral membrane; RMR, right marginal row; TC, transverse cirri. Scale bars: 20 μm (**A**–**C**, **E**) and 10 μm (**I**)

**Fig. 2:**
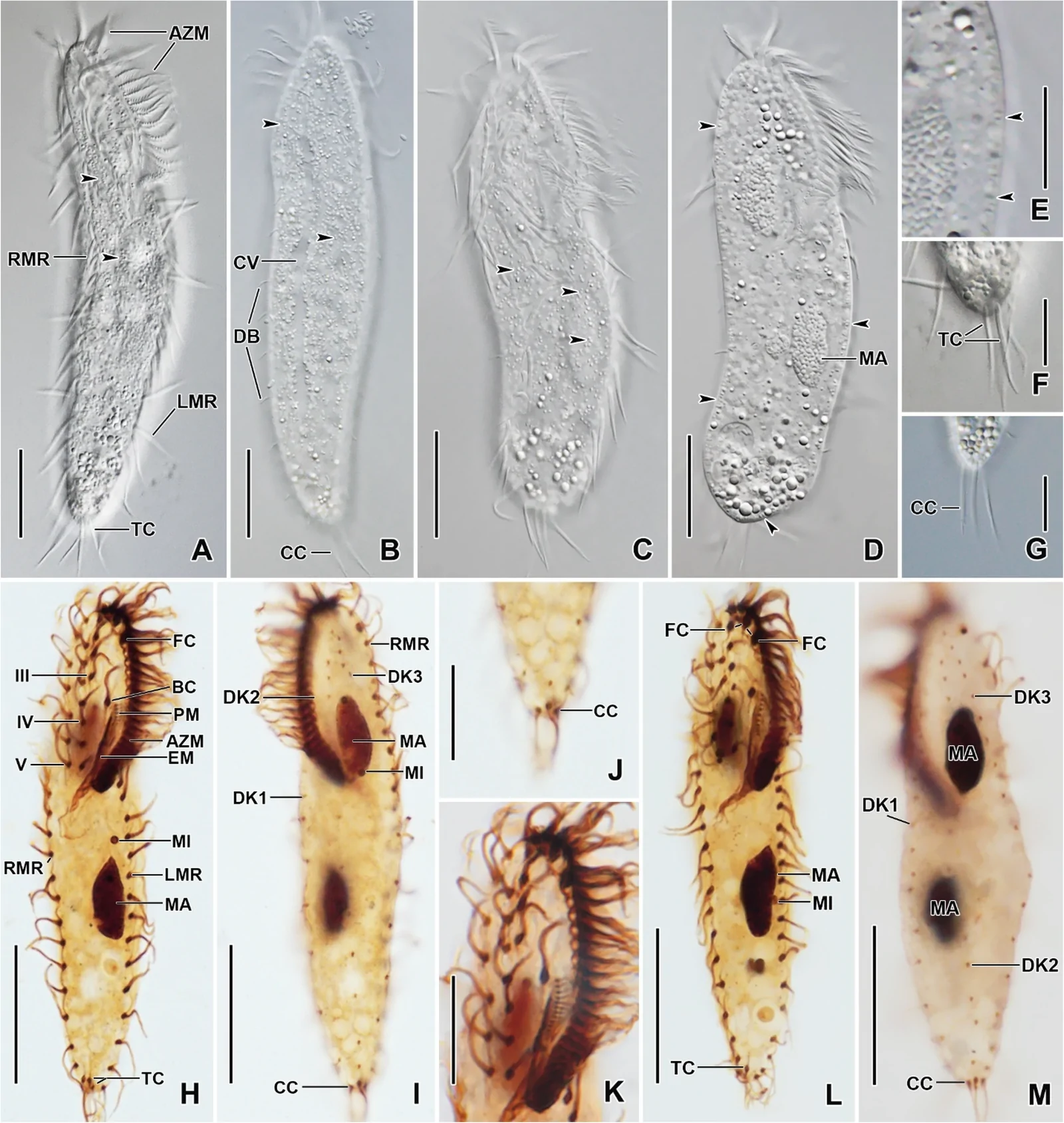
*Metagonostomoides elongatus* sp. nov., from life (**A**–**G**) and after protargol impregnation (**H**–**M**). **A–D **Ventral (**A**, **C**, **D**) and dorsal (**B**) view showing the body shape, the cirral pattern, distribution and shape of cortical granules (arrowheads), and the nuclear apparatus. **E **Optical section showing the shape of cortical granules (arrowheads). **F**,** G **Ventral (**F**) and dorsal (**G**) view of posterior end of body, showing the transverse cirri (**F**) and the caudal cirri (**G**). **H–M **Ventral (**H**, **K**, **L**) and dorsal (**I**, **J**, **M**) view of the holotype (**H**–**K**) and two paratype (**L**, **M**) specimens, showing the body shape and ciliature. AZM, adoral zone of membranelles; BC, buccal cirrus; CC, caudal cirri; CV, contractile vacuole; DB, dorsal bristles; DK1–3, dorsal kineties; EM, endoral membrane; FC, frontal cirri; FVC, frontoventral cirri; LMR, left marginal row; MA, macronuclear nodules; MI, micronuclei; PM, paroral membrane; RMR, right marginal row; TC, transverse cirri. Scale bars: 20 μm (**A**–**D**, **H**–**K**) and 10 μm (**E**–**G**, **L**, **M**)

**Table 1:** Morphometric data on *Metagonostomoides elongatus* sp. nov. (ME) and *Wallackia koreana* sp. nov. (WK)

Characteristic^a^	Species	Mean	M	SD	SE	CV	Min	Max	*n*
Body, length	ME	72.8	72.0	9.2	2.0	12.6	53.0	99.0	21
	WK	48.3	47.6	4.8	1.3	10.0	41.5	57.0	15
Body, width	ME	17.5	18.0	2.8	0.6	15.8	13.0	23.0	21
	WK	15.2	15.6	1.5	0.4	10.2	12.4	18.0	15
Body length:width, ratio	ME	4.3	4.4	1.0	0.2	22.7	2.9	7.1	21
	WK	3.2	3.3	0.3	0.1	9.0	2.5	3.6	15
Anterior body end to proximal end of AZM, distance	ME	28.7	28.0	2.5	0.6	8.8	25.0	34.0	21
	WK	29.9	29.8	2.2	0.6	7.3	25.5	33.6	15
AZM, % of body length	ME	39.8	39.7	3.7	0.8	9.3	31.3	47.2	21
	WK	62.2	61.4	4.6	1.2	7.4	53.9	71.5	15
Adoral membranelles, number	ME	23.2	23.0	1.2	0.3	5.0	21.0	26.0	21
	WK	25.7	25.0	1.8	0.5	7.2	23.0	29.0	15
Anterior body end to paroral membrane, distance	ME	13.7	14.0	2.0	0.4	14.4	11.0	18.0	21
	WK	16.4	15.9	1.5	0.4	9.4	13.8	20.0	15
Anterior body end to paroral membrane, % of body length	ME	18.8	18.9	2.0	0.4	10.8	15.6	23.2	21
	WK	34.2	33.8	3.2	0.8	9.4	27.8	41.2	15
Paroral membrane, length	ME	7.0	7.0	0.7	0.2	10.4	6.0	8.0	21
	WK	7.5	7.7	1.0	0.3	13.5	5.5	9.2	15
Paroral membrane, number of kinetids	ME	12.5	13.0	1.4	0.3	11.2	9.0	14.0	21
	WK	10.2	10.0	1.1	0.3	10.6	8.0	12.0	15
Anterior body end to endoral membrane, distance	ME	16.1	16.0	2.3	0.5	14.4	13.0	20.0	21
	WK	21.0	21.3	1.9	0.5	8.8	17.5	24.0	15
Anterior body end to endoral membrane, % of body length	ME	22.2	21.3	2.3	0.5	10.2	19.2	27.5	21
	WK	43.7	42.9	3.7	0.9	8.3	38.3	51.1	15
Endoral membrane, length	ME	11.2	11.0	1.0	0.2	9.1	10.0	14.0	21
	WK	7.1	7.0	0.9	0.2	12.5	5.5	8.6	15
Anterior body end to first macronuclear nodule, distance	ME	14.0	14.0	2.1	0.5	14.7	9.0	17.0	21
	WK	11.7	11.7	1.3	0.3	11.0	8.3	13.0	15
Nuclear figure, length	ME	38.5	36.0	5.7	1.2	14.8	30.0	52.0	21
	WK	28.5	28.4	3.3	0.9	11.7	23.0	37.0	15
Anterior macronuclear nodule, length	ME	12.7	12.0	2.2	0.5	17.4	10.0	18.0	21
	WK	11.8	12.0	1.5	0.4	13.1	8.3	13.9	15
Anterior macronuclear nodule, width	ME	5.1	5.0	0.8	0.2	15.7	4.0	6.5	21
	WK	5.0	5.0	0.4	0.1	8.3	4.0	5.5	15
Macronuclear nodules, number	ME	2.0	2.0	0.0	0.0	0.0	2.0	2.0	21
	WK	2.0	2.0	0.0	0.0	0.0	2.0	2.0	15
Macronuclear nodules, length in between	ME	12.3	12.0	2.9	0.7	23.9	8.0	18.0	20
	WK	6.1	6.0	3.2	0.8	53.3	1.0	14.8	15
Micronuclei, diameter	ME	1.0	1.0	0.0	0.0	0.0	1.0	1.0	21
	WK	2.0	2.0	0.4	0.1	22.2	1.0	2.6	15
Micronuclei, number	ME	3.0	3.0	0.8	0.2	27.3	1.0	4.0	21
	WK	1.9	2.0	0.3	0.1	13.4	1.0	2.0	15
Anterior body end to RMR, distance	ME	4.7	4.0	1.1	0.2	24.0	2.5	7.2	21
	WK	5.4	5.6	0.7	0.2	13.6	4.0	6.6	15
Anterior body end to RMR, % of body length	ME	6.4	6.0	1.4	0.3	22.0	4.0	9.7	21
	WK	11.4	12.0	2.3	0.6	19.8	8.0	15.1	15
Posterior body end to end of RMR, distance	ME	2.7	2.0	1.3	0.3	47.7	1.0	6.0	21
	WK	2.0	2.0	0.7	0.2	33.6	1.0	3.0	15
Posterior body end to end of RMR, % of body length	ME	3.7	2.9	1.8	0.4	47.1	1.0	7.6	21
	WK	4.2	4.0	1.2	0.3	28.8	2.4	6.4	15
Right marginal row, number of cirri	ME	21.0	21.0	1.2	0.3	5.7	19.0	24.0	21
	WK	13.2	14.0	1.6	0.4	11.9	11.0	15.0	15
Anterior body end to LMR, distance	ME	26.4	26.0	2.8	0.6	10.7	22.0	32.0	21
	WK	28.8	29.0	2.0	0.5	6.8	24.9	32.0	15
Anterior body end to LMR, % of body length	ME	36.5	37.0	3.1	0.7	8.4	30.3	42.0	21
	WK	59.9	59.4	4.4	1.1	7.4	51.8	68.1	15
Posterior body end to end of LMR, distance	ME	1.4	1.0	0.7	0.2	52.0	0.0	2.8	21
	WK	0.7	0.6	0.4	0.1	51.1	0.3	1.4	15
Posterior body end to end of LMR, % of body length	ME	2.0	1.4	1.1	0.2	56.2	0.0	4.4	21
	WK	1.5	1.3	0.7	0.2	44.0	0.6	2.5	15
Left marginal row, number of cirri	ME	15.7	15.0	1.5	0.3	9.5	13.0	18.0	21
	WK	10.3	11.0	1.6	0.4	15.8	7.0	12.0	15
Frontal cirri, number	ME	3.0	3.0	0.0	0.0	0.0	3.0	3.0	21
	WK	3.0	3.0	0.0	0.0	0.0	3.0	3.0	15
Anterior body end to posterior buccal cirrus, distance	ME	13.6	13.0	2.0	0.4	14.6	11.0	18.0	21
	WK	19.4	19.6	1.5	0.4	7.5	16.0	22.0	15
Buccal cirri, number	ME	1.0	1.0	0.0	0.0	0.0	1.0	1.0	21
	WK	2.0	2.0	0.0	0.0	0.0	2.0	2.0	15
Anterior body end to posterior cirrus of FV row III, distance	ME	12.9	12.0	2.1	0.5	16.3	9.0	17.0	21
	WK	18.7	19.0	2.6	0.7	13.7	14.9	23.0	15
Frontoventral cirral row III, number of cirri^b^	ME	3.2	3.0	0.4	0.1	12.6	3.0	4.0	21
	WK	3.8	4.0	0.4	0.1	10.9	3.0	4.0	15
Anterior body end to anterior cirrus of FV row IV, distance	ME	6.9	7.0	1.1	0.2	16.4	5.0	9.0	21
	WK	7.9	8.2	1.1	0.3	14.3	5.4	9.5	15
Anterior body end to posterior cirrus of FV row IV, distance	ME	26.3	26.0	3.3	0.7	12.6	20.0	34.0	21
	WK	46.1	46.0	4.8	1.2	10.5	38.3	55.0	15
Anterior body end to end of FV row IV, % of body length	ME	36.4	36.8	4.3	0.9	11.8	26.3	46.4	21
	WK	95.4	95.7	1.9	0.5	2.0	92.3	97.8	15
Frontoventral cirral row IV, number of cirri	ME	6.7	7.0	0.8	0.2	11.9	5.0	8.0	21
	WK	13.9	14.0	1.4	0.4	10.1	11.0	16.0	15
Anterior body end to anterior cirrus of FV row V, distance	ME	2.6	2.5	0.8	0.2	29.8	1.5	4.7	21
	WK	3.9	3.9	0.9	0.2	23.8	2.4	6.0	15
Anterior body end to posterior cirrus of FV row V, distance	ME	28.9	29.0	4.2	0.9	14.5	21.0	40.0	21
	WK	47.2	46.0	4.6	1.2	9.7	40.8	56.0	15
Anterior body end to end of FV row V, % of body length	ME	39.9	41.6	4.8	1.1	12.1	30.3	47.6	21
	WK	97.7	97.9	2.0	0.5	2.1	92.2	100.0	15
Frontoventral cirral row V, number of cirri	ME	8.4	8.0	1.0	0.2	11.6	7.0	10.0	21
	WK	15.2	16.0	1.6	0.4	10.6	11.0	17.0	15
Transverse cirri, number	ME	1.7	2.0	0.5	0.1	27.0	1.0	2.0	21
Posterior body end to transverse cirri, distance	ME	1.4	1.0	0.5	0.1	34.6	1.0	2.0	21
Frontal-ventral-transverse cirri, total number	ME	24.0	24.0	2.1	0.5	8.6	20.0	28.0	21
	WK	37.9	39.0	3.0	0.8	7.9	30.0	41.0	15
Dorsal kineties, number	ME	3.0	3.0	0.0	0.0	0.0	3.0	3.0	21
	WK	3.0	3.0	0.0	0.0	0.0	3.0	3.0	15
Anterior body end to anterior end of DK1, distance	ME	14.5	14.0	2.5	0.5	17.2	10.0	19.0	21
	WK	9.3	9.0	1.9	0.5	20.6	7.0	13.0	15
Dorsal kinety 1, number of bristles	ME	11.4	11.0	1.2	0.3	10.6	10.0	14.0	21
	WK	16.1	16.0	2.2	0.6	13.6	12.0	20.0	15
Anterior body end to anterior end of DK2, distance	ME	7.7	7.7	2.2	0.5	27.9	5.0	14.0	21
	WK	4.8	5.0	0.9	0.2	19.5	3.0	6.4	15
Dorsal kinety 2, number of bristles	ME	13.7	14.0	1.4	0.3	10.3	11.0	18.0	21
	WK	15.5	15.0	2.2	0.6	14.4	11.0	21.0	15
Anterior body end to anterior end of DK3, distance	ME	4.0	4.0	0.9	0.2	21.4	3.0	6.0	21
	WK	4.2	4.3	1.1	0.3	26.6	2.4	7.0	15
Dorsal kinety 3, number of bristles	ME	15.5	15.0	1.5	0.3	9.9	12.0	19.0	21
	WK	16.2	16.0	1.8	0.5	11.0	12.0	19.0	15
Dorsal bristles, total number	ME	40.6	40.0	3.1	0.7	7.6	36.0	48.0	21
	WK	47.9	48.0	5.2	1.3	10.8	35.0	57.0	15
Caudal cirri, number	ME	3.0	3.0	0.0	0.0	0.0	3.0	3.0	21
	WK	3.0	3.0	0.0	0.0	0.0	3.0	3.0	15

^a^ Data based on protargol-impregnated, randomly selected specimens. Measurements in µm. AZM, adoral zone of membranelles; CV, coefficient of variation in %; DK, dorsal kineties; FV, frontoventral cirral; LMR, left marginal row; M, median; Max, maximum; Mean, arithmetic mean; Min, minimum; *n*, number of individuals investigated; RMR, right marginal row; SD, standard deviation; SE, standard error of arithmetic mean

^b^ Rightmost frontal cirrus not included

#### ZooBank registration: urn:lsid:zoobank.org:act:87FC8AE2-A143-49B5-8C38-B47F92A9F281

#### Diagnosis

Body size 71–110 × 18–25 µm in vivo and 53–99 × 13–23 µm after protargol impregnation. Body cultrate to very narrowly ellipsoidal with posterior end narrower than anterior. Two macronuclear nodules and one to four micronuclei. Right marginal row composed of 19–24 cirri, left row of 13–18 cirri. Adoral zone occupies 31%–47% of body length and is composed of 21–26 membranelles.

#### Type locality

Soil from Cheongdae Mountain, Sokcho-si, Gangwon-do, South Korea (38°10′35.20″N, 128°34′43.20″E).

#### Type material

The protargol-impregnated slide (NNIBRPR28047) containing the holotype specimen (Figs. 1B–D and 2H–K) and one paratype slide (NNIBRPR28048) with protargol-impregnated specimens have been deposited in the Nakdonggang National Institute of Biological Resources (NNIBR), South Korea. Five other paratype slides (GUC004297, GUC004303, GUC004326–GUC004328) have been deposited in the Jung Laboratory (J.-H. Jung) at Kangwon National University, Korea. The holotype and paratypes have been marked by black ink circles on the back of the slides.

#### Etymology

The species-group name *elongatus*, -*a*, -*um* (Latin adjective [m; f; n]; elongated, stretched) refers to body outline.

#### Description

Size 71–110 × 18–25 μm in vivo (usually 89 × 21 μm, *n* = 5); 53–99 × 13–23 μm after protargol impregnation (on average 73 × 18 μm). Body slightly flexible, not contractile; cultrate to very narrowly ellipsoidal, posterior end narrower than anterior; length: width ratio 2.9–7.1:1 (4.3:1 on average) after protargol impregnation; left and right margins slightly convex; dorsoventrally flattened up to 2:1 (Figs. 1A–C and E–H and 2A–D and H–K). Two ellipsoidal macronuclear nodules separated by distance of about 12 μm; each macronucleus about 12.7 × 5.1 μm in protargol preparations. One to four micronuclei (three on average), globular, near or attached to macronuclear nodules at various positions; each about 1 μm in diameter after protargol impregnation (Figs. 1A and C and 2D and H–K). Contractile vacuole near the left margin, slightly anterior to mid-body; about 9 μm across at end-diastole; collecting canals extend into second and third quarters of cell (Figs. 1A and E–H and 2B). Cortical granules colorless, circular in cross section and rod-shaped in longitudinal view; randomly distributed over cell surface; about 1.2–1.6 × 0.3–0.5 μm (Figs. 1E and I and 2A–E). Cytoplasm colorless; food vacuoles 5–8 μm across with bacteria; refractive globular granules (likely lipid droplets) 1.0–1.7 μm across (Fig. 2C–E). Feeds on bacteria. Swims rapidly, rotating around main body axis; occasionally crawls on the bottom of the culture dish.

Adoral zone gonostomatid; occupies about 40% of body length; composed of 23 membranelles on average; cilia about 13 μm long in vivo. Buccal cavity narrow, flat. Undulating membranes parallel. Paroral membrane commences at about 19% of body length and about 2 μm anterior to endoral; extends along left side of anterior half of the endoral; composed of 9–14 widely spaced kinetids; cilia about 9 μm long in vivo. Endoral 10–14 μm long, kinetids densely spaced (Figs. 1A, B and D and 2H, J and M).

Three frontal cirri invariably; cilia about 13 μm long in vivo; left frontal cirrus posterior to level of middle and right frontal cirri. Single buccal cirrus right of distal end of the paroral. Frontoventral cirral row III usually with three or four parabuccal cirri posterior to right frontal cirrus. Frontoventral cirral row IV begins to the right of, and slightly posterior to, the first parabuccal cirrus; consists of 5–8 cirri; terminates at 26–46% (36% on average) of body length. Frontoventral cirral row V begins to the right of, and at level of, right frontal cirrus, consists of 7–10 cirri; extends over 30–48% (40% on average) of body length. Frontoventral cirri cilia about 10 μm long in vivo. One or two transverse cirri left of posteriormost right marginal cirrus; cilia about 10 μm long in vivo (Figs. 1A–C and 2A, C, F, H, J and M). Right marginal row begins at about 6% of body length and ends at about 96%; anterior portion on lateral or dorsal side; consists of 19–24 cirri. Left marginal row begins at about 37% of body length and ends at about 98%; consisting of 13–18 cirri; right and left marginal rows never join posteriorly. Marginal cirri similar in size to frontoventral cirri in vivo (Figs. 1A and B and 2A, H and J).

Three dorsal kineties; composed of 10–14, 11–18, and 12–19 dikinetids, respectively; kineties 1 and 3 slightly shortened anteriorly. Dorsal bristles 2.7–3.5 μm long in vivo. Each dorsal kinety terminates with a single caudal cirrus; cilia about 15 μm long in vivo (Figs. 1A and C and 2G, I, K and L).

### Morphogenesis of *Metagonostomoides elongatus* sp. nov. (Figs. 3, 4, 5 and 6)

**Fig. 3:**
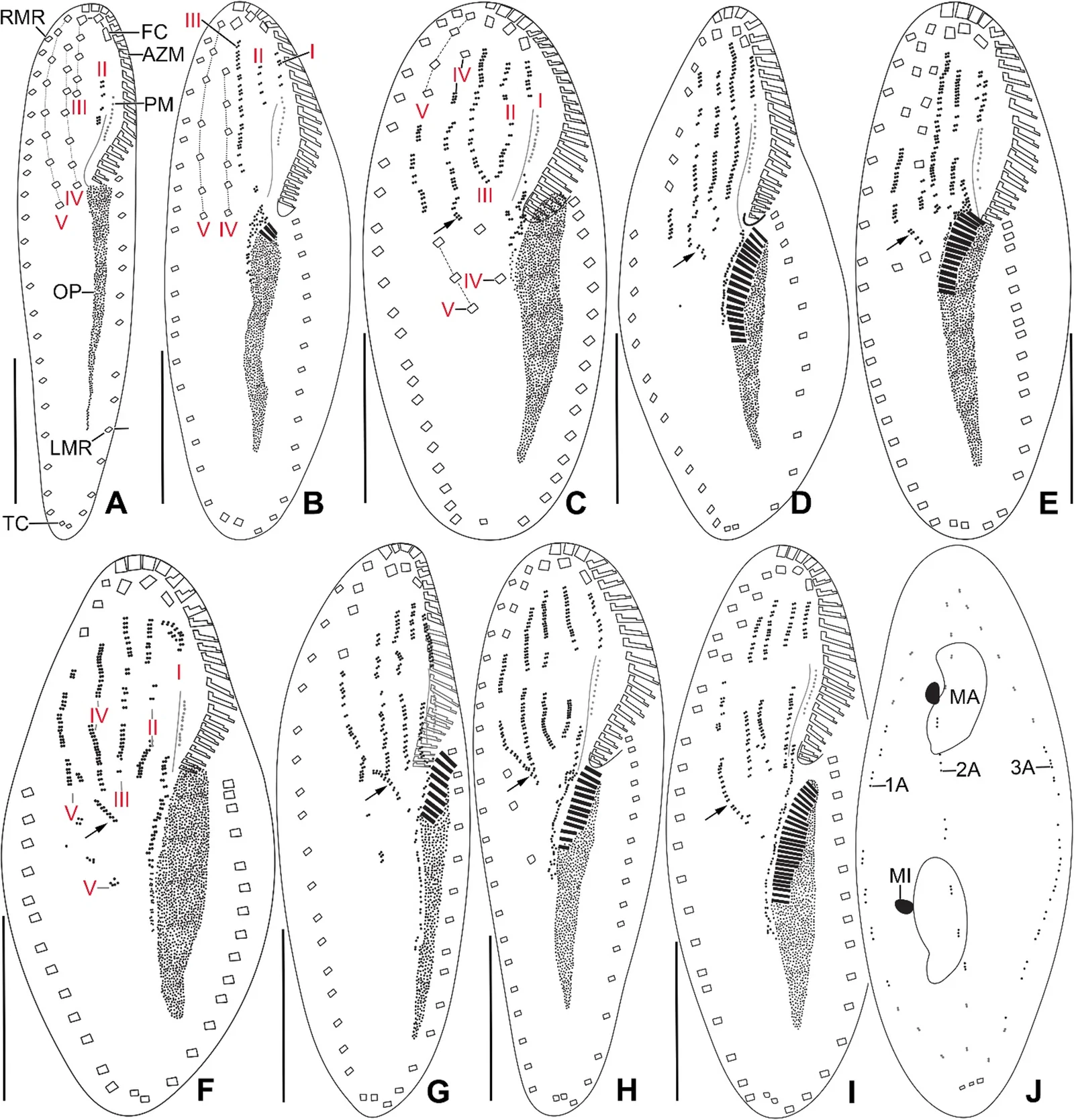
*Metagonostomoides elongatus* sp. nov., morphogenesis after protargol impregnation. **A** A very early divider showing the formation of oral primordium and the disaggregation of buccal cirrus. **B**, **C** Early dividers, showing the de novo origin of anlage I (UMA) ahead of parental undulating membranes (**B**) and the dedifferentiation of frontoventral cirral rows III–V to form primary primordia III–V (**C**). **D**–**I **Early dividers, showing the separation of the posterior portion of primary primordium IV and connects with the posterior end of primary primordium V (arrows) and the transverse division of primary primordia II–V in the middle to form secondary primordia in each daughter cell. **J** Dorsal view belongs to the specimen in (**I**) showing the origin of dorsal kineties anlagen at one level in dorsal kineties. 1A–3A, dorsal kineties anlagen; AZM, adoral zone of membranelles; FC, frontal cirri; I–V, frontoventral cirral rows and primordia; LMR, left marginal row; MA, macronuclear nodule; MI, micronucleus; OP, oral primordium; PM, paroral membrane; RMR, right marginal row; TC, transverse cirri. Scale bars: 20 μm

**Fig. 4:**
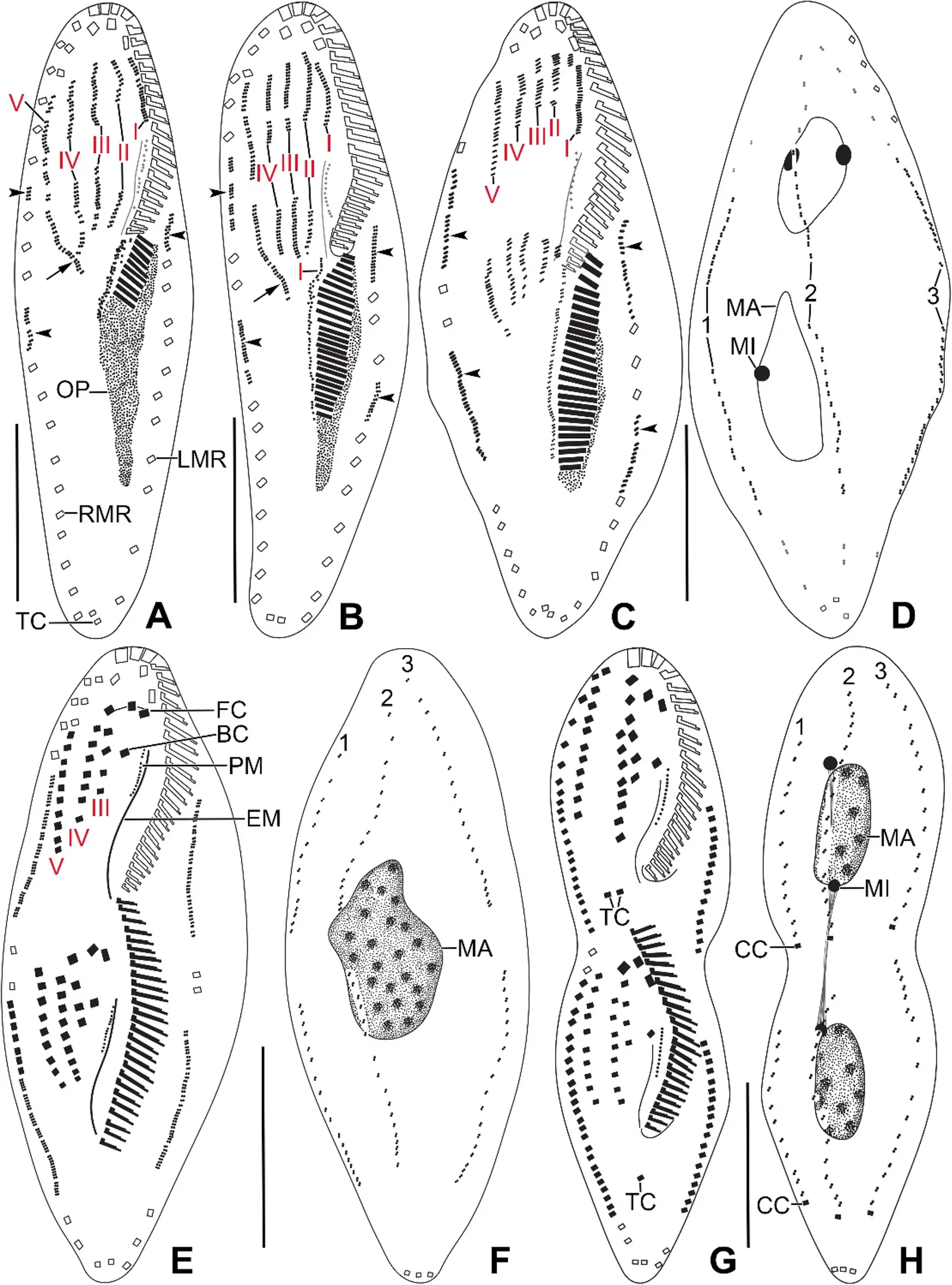
*Metagonostomoides elongatus* sp. nov., morphogenesis after protargol impregnation. **A**, **B**Early dividers showing the fusion of the posterior portion of primordium IV to the posterior end of primordium V (arrows) and the origin of marginal anlagen at two levels within the marginal cirral rows (arrowheads). **C**, **D **Ventral (**C**) and dorsal (**D**) view of early middle divider, showing the secondary primordia in each daughter cell, the marginal cirral anlagen (arrowheads), and the dorsal anlagen. **E**, **F **Ventral (**E**) and dorsal (**F**) view of a middle divider, showing the differentiation of primordia into cirri, and the fusion of the macronuclear nodules. **G**, **H **Ventral (**G**) and dorsal (**H**) view of late divider, showing the migration of cirri to their final locations and the formation of new caudal cirri. 1–3, dorsal kineties; BC, buccal cirri; CC, caudal cirri, EM, endoral membrane; FC, frontal cirri; I–V, secondary primordia; LMR, left marginal row; MA, macronuclear nodule; MI, micronucleus; OP, oral primordium; PM, paroral membrane; RMR, right marginal row; TC, transverse cirri. Scale bars: 20 μm

**Fig. 5:**
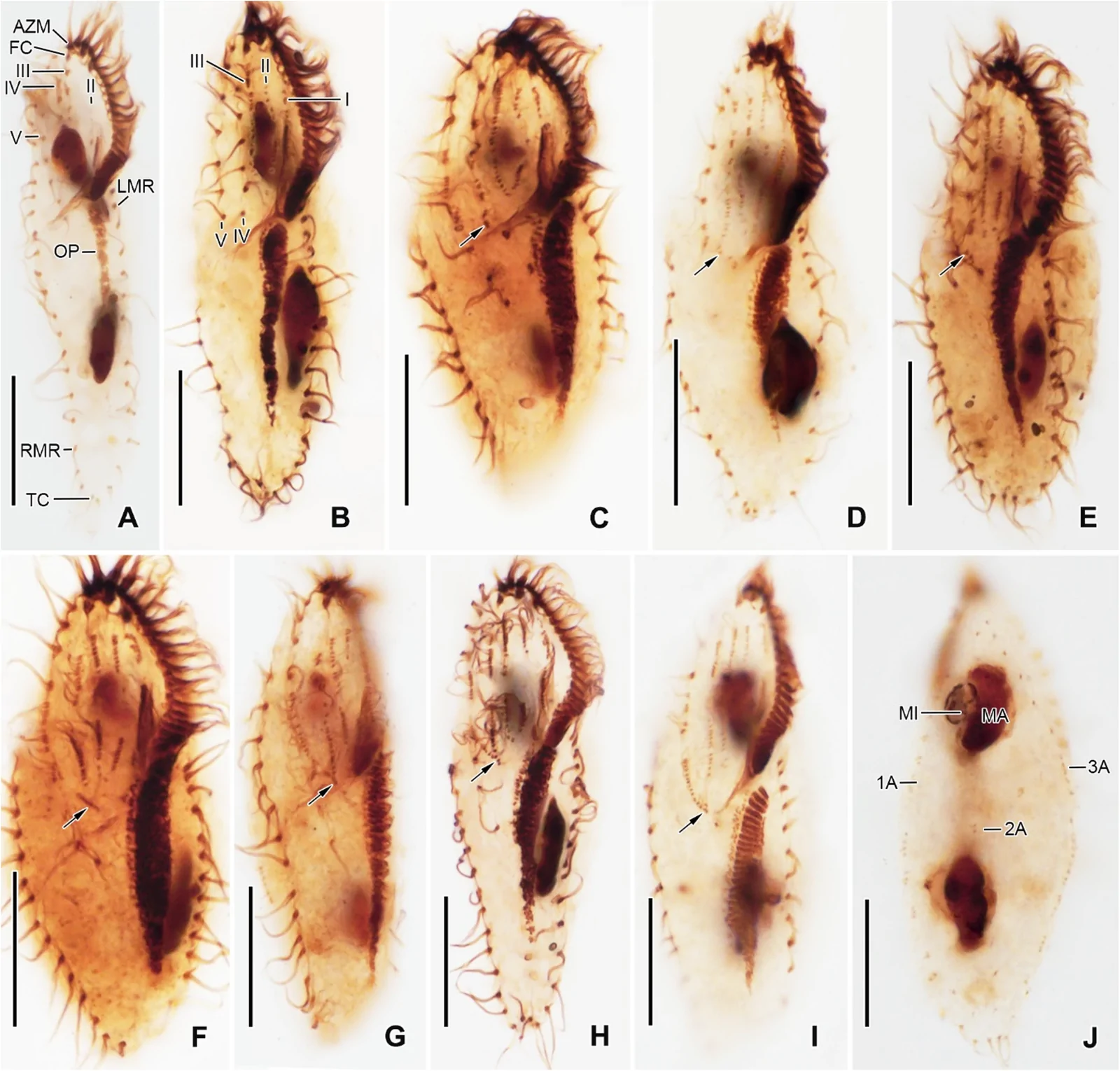
*Metagonostomoides elongatus* sp. nov., morphogenesis after protargol impregnation. **A** A very early divider showing the formation of oral primordium and the disaggregation of buccal cirrus. **B**, **C** Early dividers, showing the de novo origin of anlage I (UMA) ahead of parental undulating membranes (**B**) and the dedifferentiation of frontoventral cirral rows III–V to form primary primordia III–V (**C**). **D**–**I** Early dividers, showing the posterior portion of primary primordium IV separates and merges with the posterior end of primary primordium V (arrows) and the transverse division of primary primordia II–V in the middle to form secondary primordia in each daughter cell. **J** Dorsal view belongs to the specimen in (**I**) showing the origin of dorsal kineties anlagen at one level in dorsal kineties. 1A–3A, dorsal kineties anlagen; AZM, adoral zone of membranelles; FC, frontal cirri; I–V, frontoventral cirral rows and primordia; LMR, left marginal row; MA, macronuclear nodule; MI, micronucleus; OP, oral primordium; PM, paroral membrane; RMR, right marginal row; TC, transverse cirri. Scale bars: 20 μm.

**Fig. 6:**
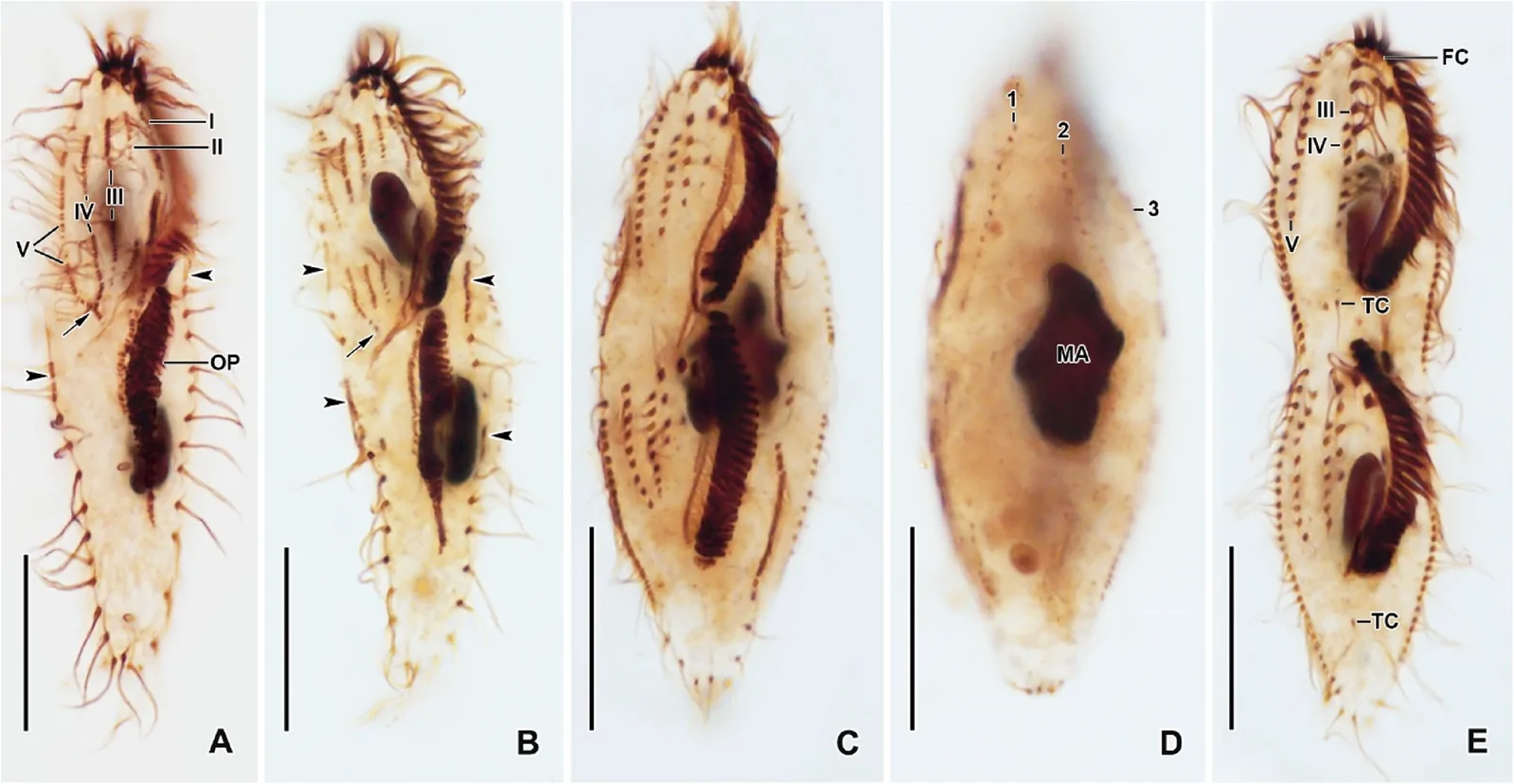
*Metagonostomoides elongatus* sp. nov., morphogenesis after protargol impregnation. **A**, **B **Early dividers showing the fusion of the posterior portion of primordium IV to the posterior end of primordium V (arrows) and the origin of marginal anlagen at two levels within the marginal cirral rows (arrowheads). **C**, **D **Ventral (**C**) and dorsal (**D**) view of a middle divider, showing the differentiation of primordia into cirri, and the fusion of the macronuclear nodules. **E **Ventral view of late divider, showing the migration of cirri to their final locations. 1–3, dorsal kineties; FC, frontal cirri; I–V, secondary primordia; MA, macronuclear nodule; OP, oral primordium; TC, transverse cirri. Scale bars: 20 μm

#### Stomatogenesis

Cell division begins with the apokinetal formation of a streak of basal bodies on the cell surface posterior to the buccal vertex and to the right of the left marginal row. These basal bodies proliferate to form the oral primordium (Figs. 3A and 5A). Next, a line of dikinetids appears de novo anterior to parental paroral membrane, forming anlage I (UMA, undulating membranes anlage) of the proter. At the same time, new adoral membranelles are formed at the anterior right side of the oral primordium, and anlage I of the opisthe appears as a streak of basal bodies to the right of the oral primordium (Figs. 3B and 5B). In middle dividers, the UMA of the proter and opisthe produce the left frontal cirrus and the new undulating membranes of the proter and opisthe, respectively (Figs. 4E and 6C). The parental undulating membranes are highly likely resorbed in the next stage without contributing to the new undulating membranes formation (Figs. 4E and 6C). The parental adoral membranelles are completely retained by the proter.

#### Development of frontal-ventral-transverse cirri

All frontoventral anlagen II–V arise as primary primordia and then split in the middle to form secondary primordia for each daughter cell (Figs. 3A–I and 5A–I). In very early dividers, primary primordium II is formed by the disaggregation and elongation of the buccal cirrus (Figs. 3A and B and 5A and B). Shortly thereafter, all cirri of frontoventral cirral row III, except the right frontal cirrus, disaggregate and form primary primordium III (Figs. 3B and 5B). Next, the middle cirri of each of frontoventral cirral rows IV and V disaggregate, forming primary primordia IV and V, respectively. Both primary primordia elongate anteriorly by incorporating additional parental cirri. Primary primordium IV very likely also extends posteriorly by incorporating a few posterior cirri of row IV (Figs. 3C–F and 5C–F). The remaining posterior parental cirri of frontoventral cirral row V gradually disaggregate and disappear; in some cells these cirri disappear faster than in others (Figs. 3C–H and 5C–H). The posterior portion of primary primordium IV becomes obliquely arranged, separates from the remainder of primary primordium IV, and merges with the posterior end of primary primordium V (Figs. 3C–J, 4A and B, 5C–J and 6A and B). Primary primordia II, III, IV, and the rightmost primary primordium (V), which incorporates the posterior portion of primary primordium IV together with the entire primary primordium V, then split in the middle to form secondary primordia (Figs. 3F–I, 4A–C, 5F–I and 6A and B). The anterior secondary primordia remain in the proter, while the posterior secondary primordia migrate posteriorly to form the frontoventral cirral rows of the opisthe (Figs. 3H and I and 5H and I). In middle dividers, all primordia differentiate into cirri (Figs. 4E and 6C). In late dividers, these cirri migrate to their final positions (Figs. 4G and 6E). The posterior cirrus of each frontoventral cirral row IV and V, or only that of one of the rows, migrates posteriorly to form either two or one transverse cirrus, respectively (Figs. 4G and 6E).

#### Development of the marginal cirral rows

The right marginal anlagen and the anterior left marginal anlage originate intrakinetally in early stages of division (Figs. 4A and 6A). The anterior right marginal anlage arises by disintegration of the fifth or sixth right marginal cirrus and elongates posteriorly by dedifferentiation of parental cirri (Figs. 4B and C and 6B and C). The posterior right marginal anlage appears slightly posterior to mid-body and also extends posteriorly within the row (Figs. 4B and C and 6B and C). The anterior left marginal anlage arises at the anterior end of the left marginal row. The posterior left marginal anlage appears shortly afterward, posterior to mid-body and separated from the anterior anlage by two or three parental cirri, and extends posteriorly by dedifferentiation of parental cirri (Figs. 4A–C and 6A–C). In the late dividers, all marginal anlagen differentiate into cirri (Figs. 4G and 6E).

#### Development of dorsal kineties

Dorsal kinety anlagen appear in early stages and develop intrakinetally in the middle portion of dorsal kineties 1–3. Initially, the dikinetids separate and become widely spaced; some of them become trikinetids by insertion of a new kinetid between the separating pair (Figs. 3J and 5J). Subsequently, a new basal body is formed posterior to each kinetid (Figs. 4D and F and 6D). Caudal cirri appear very late in division, one at the posterior end of each kinety (Fig. 4H).

#### Division of nuclear apparatus

The two macronuclear nodules fuse into a single mass in late stages (Figs. 4F and 6D). This mass elongates and divides into one nodule for each daughter cell in very late stages (Figs. 4H and 6E). Shortly before daughter cell separation, each macronuclear nodule likely divides again to reconstitute two macronuclear nodules per cell. Micronuclei divide by mitosis.

### Family Wallackiidae fam. nov.

#### ZooBank registration: urn:lsid:zoobank.org:act:88506C4E-8DFA-4433-9A67-8F3AF6AC47F7

#### Diagnosis

Non-dorsomarginalian hypotrichs with a gonostomatid oral apparatus and five frontoventral cirral rows/anlagen, including a buccal cirral row with at least two cirri. During morphogenesis, proter and opisthe form their FVT anlagen independently (no shared primary primordia). In the opisthe, anlagen I–III arise from the oral primordium and remain separate from the corresponding proter anlagen. Anlagen IV and V arise intrakinetally at two distinct levels, sometimes without intervening parental cirri, but never as continuous primordia. Transverse cirri sensu stricto absent. Three dorsal kineties with or without caudal cirri.

#### Type genus

*Wallackia* Foissner, 1976.

#### Genera included

*Wallackia* Foissner, 1976 and *Neowallackia* Berger, 2011.

### *Wallackia* Foissner, 1976

#### Improved diagnosis

Wallackiidae with one right and one left marginal cirral row; caudal cirri present, with a single caudal cirrus at the posterior end of each dorsal kinety.

### *Wallackia koreana* sp. nov. (Figs. 7 and 8; Table 1)

**Fig. 7:**
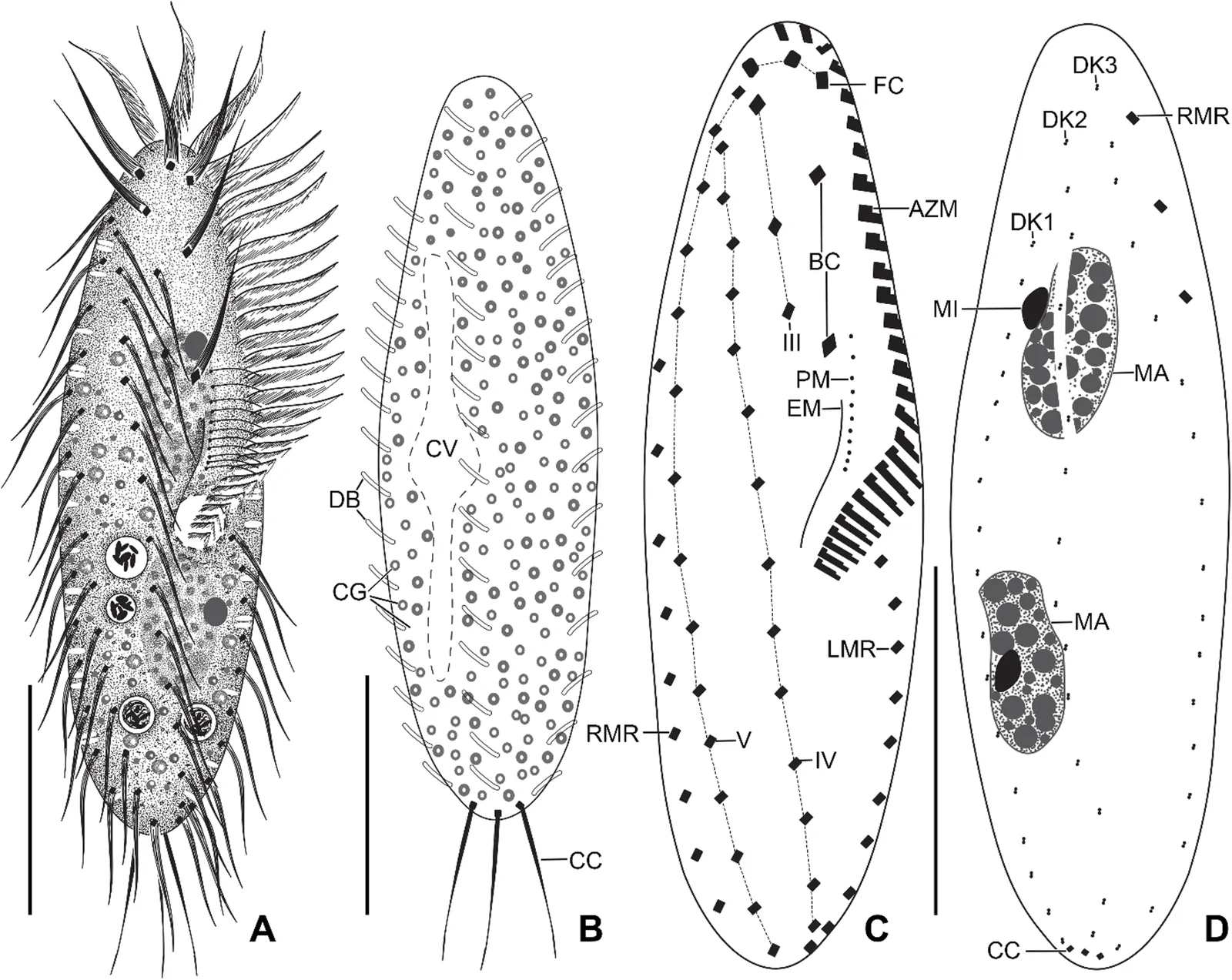
*Wallackia koreana* sp. nov., from life (**A**, **B**) and after protargol impregnation (**C**, **D**). **A **Ventral view of a representative specimen, showing, inter alia, the body shape and the cirral pattern. **B** Dorsal view showing the arrangement of the cortical granules, the caudal cirri, the contractile vacuole, and the dorsal bristles. **C**, **D **Ventral (**C**) and dorsal (**D**) view of the holotype specimen, showing the cirral pattern including two buccal cirri, the undulating membranes in *Gonostomum* pattern, the nuclear apparatus, the three dorsal kineties, and the fine caudal cirri. AZM, adoral zone of membranelles; BC, buccal cirri; CC, caudal cirri; CG, cortical granules; CV, contractile vacuole; DB, dorsal bristles; DK1–3, dorsal kineties; EM, endoral membrane; FC, frontal cirri; III–V, frontoventral cirral rows; LMR, left marginal row; MA, macronuclear nodules; MI, micronucleus; PM, paroral membrane; RMR, right marginal row. Scale bars: 20 μm

**Fig. 8:**
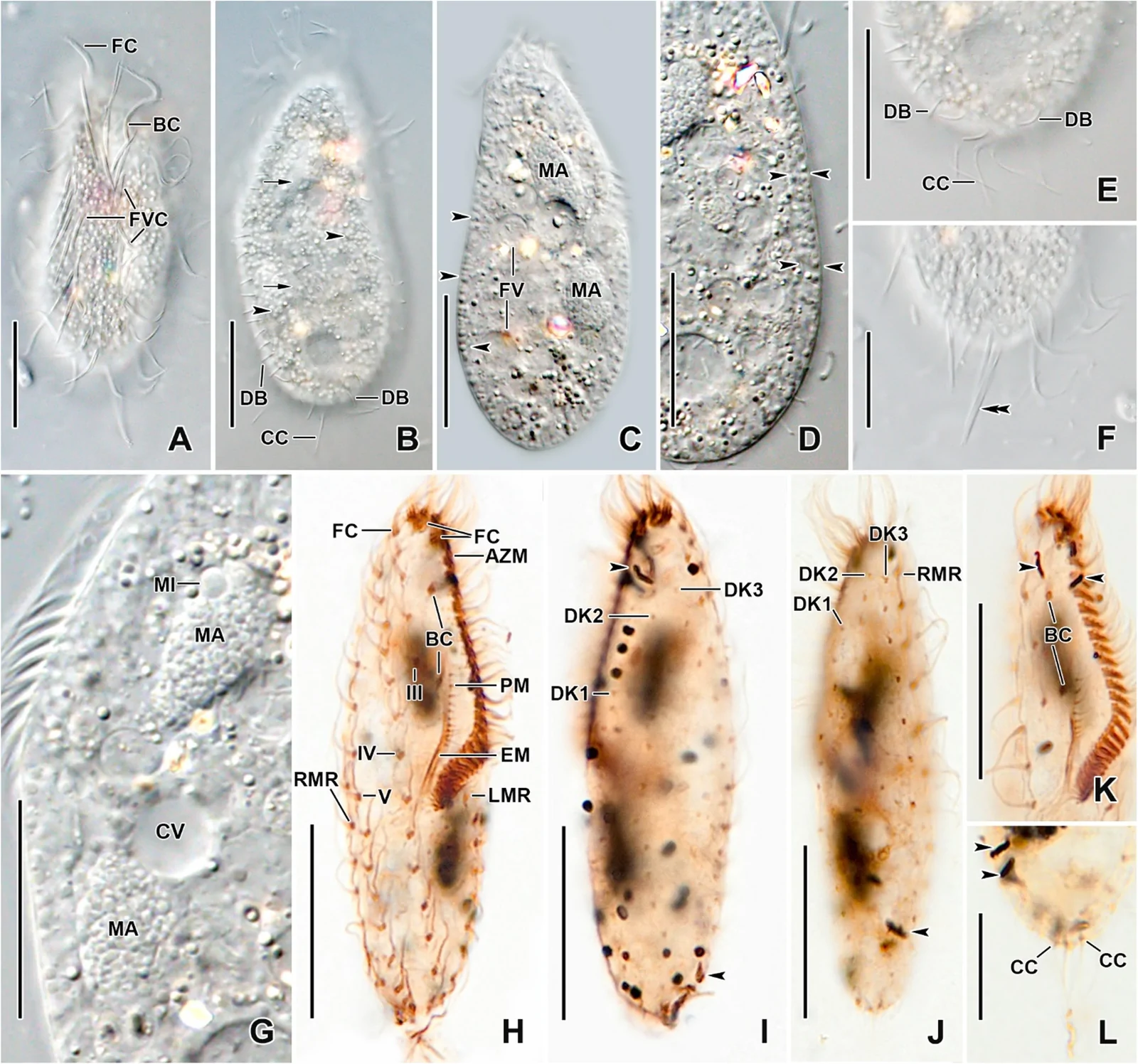
*Wallackia koreana* sp. nov., from life (**A**–**G**) and after protargol impregnation (**H**–**L**). **A**–**C **Ventral (**A**, **B**) and dorsal (**C**) view, showing the body shape, the cirral pattern, distribution and shape of cortical granules (arrowheads), and the nuclear apparatus. **D** Optical section showing the cortical granules forming seam (opposite arrowheads). **E** Dorsal view of the posterior end of body, showing the fine caudal cirri and the dorsal bristles. **F** Ventral view of posterior end of body, showing the enlarged posterior cirri in rows IV and V (double arrowheads). **G** Optical section showing the nuclear apparatus and the contractile vacuole. **H**–**L** Ventral (**H**, **K**) and dorsal (**I**, **J**, **L**) views of the holotype (**H**, **I**) and paratype (**J**–**L**) specimens, showing the body shape and ciliature. Arrowheads mark the cortical granules. AZM, adoral zone of membranelles; BC, buccal cirri; CC, caudal cirri; CV, contractile vacuole; DB, dorsal bristles; DK1–3, dorsal kineties; EM, endoral membrane; FC, frontal cirri; FV, food vacuoles, FVC, frontoventral cirri; III–V, frontoventral cirral rows; LMR, left marginal row; MA, macronuclear nodules; MI, micronucleus; PM, paroral membrane; RMR, right marginal row. Scale bars: 20 μm (**A**–**K**) and 10 μm (**L**)

#### ZooBank registration: urn:lsid:zoobank.org:act:F0BCA1D2-0DC2-4ACA-98B7-CC7612E1712C

#### Diagnosis

Body size 50–70 × 15–23 μm in vivo and 42–57 × 12–18 μm after protargol impregnation. Body ellipsoid. Two macronuclear nodules and two, rarely one, micronucleus. Cortical granules colorless and ellipsoidal to broadly ellipsoidal. Two buccal cirri, frontoventral cirral row III with three or four cirri, row IV with 11–16 cirri, row V with 11–17 cirri. Right marginal row composed of 11–15 cirri, left row of 7–12 cirri. Adoral zone occupies 54%–72% of body length and is composed of 23–29 membranelles.

#### Type locality

Temporary puddle on a grass lawn in Kangwon National University, Gangneung, South Korea (37°46’12.4’’N, 128°52’16.5’’E).

#### Type material

The slide (NNIBRPR28049) containing the holotype (Figs. 7C and D and 8H and I) and one paratype slide (NNIBRPR28050) with protargol-impregnated specimens have been deposited in the Nakdonggang National Institute of Biological Resources (NNIBR), South Korea. Two other paratype slides (GUC003644 and GUC003645) have been deposited in the Jung Laboratory (J.-H. Jung) at Kangwon National University, Korea. The holotype and paratypes have been marked by black ink circles on the back of the slides.

#### Etymology

The species-group name ‘*koreana’* refers to the country where the species was discovered.

#### Description

Size 50–70 × 15–23 μm in vivo (usually 60 × 18 μm, *n* = 5); 42–57 × 12–18 μm after protargol impregnation (on average 48 × 15 μm). Body slightly flexible, not contractile; ellipsoidal; length: width ratio 2.5–3.6:1 (3.2:1 on average) after protargol impregnation; margins slightly convex; dorsoventrally flattened up to 2:1 (Figs. 7A–D and 8A–D and H–J). Nuclear apparatus left of midline, two ellipsoidal macronuclear nodules separated by distance of about 6 μm; each macronucleus about 11.8 × 5.0 μm in protargol preparations. Two (rarely one) micronuclei, globular to fusiform, attached to macronuclear nodules at various positions; each about 2 μm in diameter after protargol impregnation (Figs. 7A and D and 8D, G and I). Contractile vacuole between left margin and body’s midline, slightly at mid-body; about 7 μm across at end-diastole; collecting canals indistinct (Figs. 7A and B and 8C and G). Cortical granules (extrusomes) colorless, ellipsoidal to broadly ellipsoidal; numerous, randomly distributed over cell surface, forming a conspicuous seam; about 1.8–2.0 × 1.0–1.5 μm (Figs. 7A and B and 8B–F). Cytoplasm colorless, food vacuoles with bacteria; refractive globular granules (likely lipid droplets) present (Fig. 8A–G). Feeds on bacteria. Usually crawls on the bottom of the culture dish.

Adoral zone gonostomatid, occupies about 62% of body length; composed of 26 membranelles on average; cilia about 15 μm long in vivo. Buccal cavity narrow and flat. Undulating membranes parallel. Paroral membrane about 7.5 μm long; commences at about 34% of body length and about 5 μm anterior to endoral; extends along left side of anterior half of endoral; composed of 8–12 widely spaced kinetids; cilia about 8 μm long in vivo. Endoral about 7 μm long, kinetids densely spaced (Figs. 7A and C and 8H and K).

Invariably three frontal cirri, with cilia about 15 μm long in vivo. Two buccal cirri with cilia about 15 μm long in vivo; anterior buccal cirrus about 7 μm anterior to paroral; posterior cirrus right of anterior end of paroral. Frontoventral cirral row III usually with three or four cirri posterior to right frontal cirrus; terminates at about 39% of body length. Frontoventral cirral row IV begins posterior to level of first cirrus of row III, consists of 11–16 cirri; ends near posterior body end. Frontoventral cirral row V begins right of and posterior to level of right frontal cirrus, consists of 11–17 cirri; extends to near posterior body end. Frontoventral cirri with cilia about 12 μm long in vivo, except posteriormost cirrus of row IV with cilia about 15 μm long (Figs. 7A, C and D and 8A, F, H, J and K). Right marginal row begins at about 11% of body length and ends near posterior body end; anterior portion lies on dorsal side; consists of 11–15 cirri. Left marginal row begins at about 60% of body length and ends at posterior end; consisting of 7–12 cirri; marginal cirri similar in size to frontoventral cirri in vivo (Figs. 7A, C and D and 8A, F, H, J and K).

Three dorsal kineties; composed of 12–20, 11–21, and 12–19 dikinetids, respectively; kinety 1 slightly to distinctly shortened anteriorly. Dorsal bristles about 3.7 μm long in vivo. Each dorsal kinety terminates posteriorly in a single caudal cirrus; caudal cirri fine and tapering distally (eyelash-like), without a recognizable “Pasteur pipette” shape; cilia about 15 μm long in vivo (Figs. 7B and D and 8B, E, I, J and L).

#### Molecular characterization and phylogenetic analyses

**Fig. 9:**
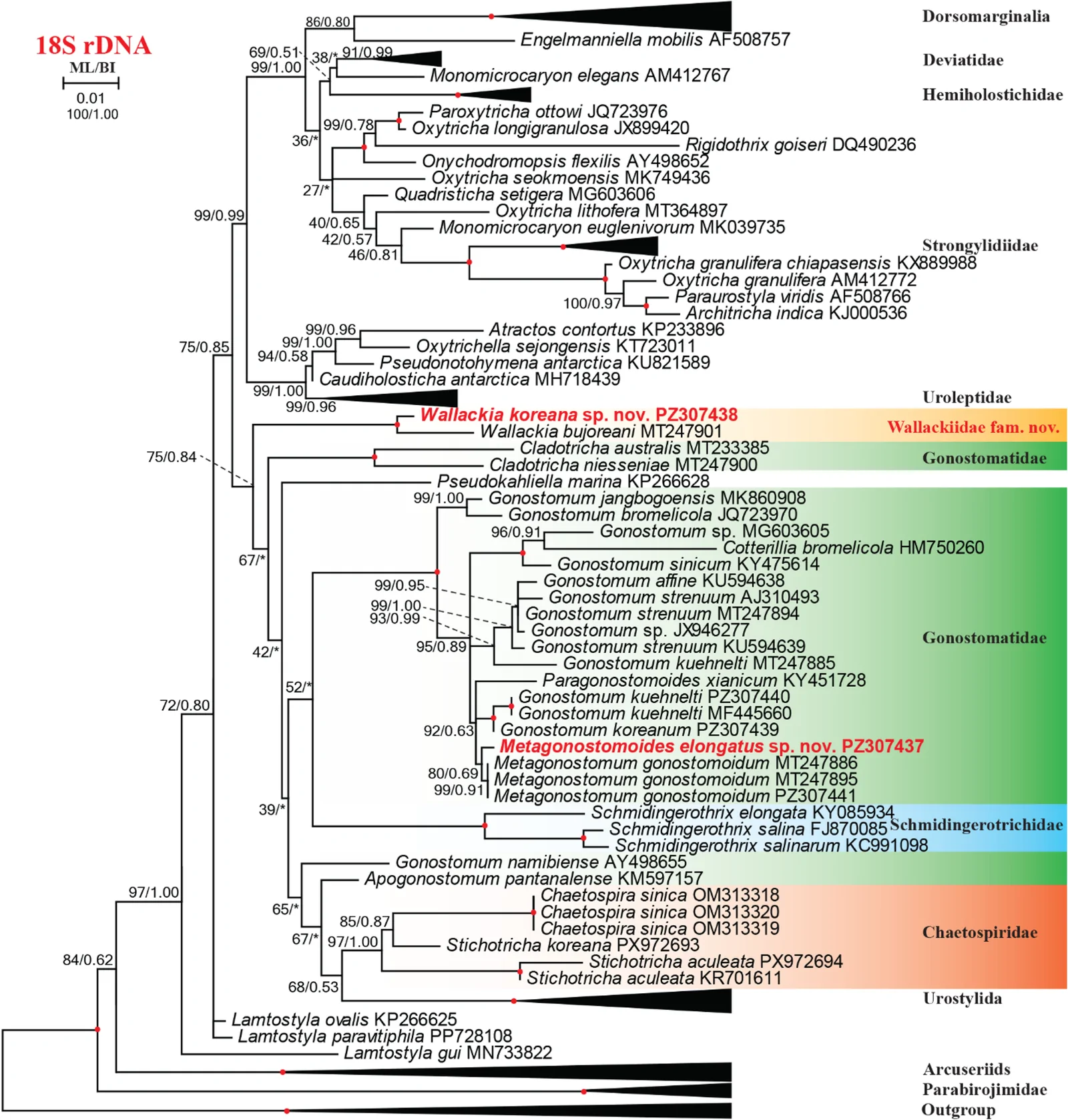
Maximum likelihood (ML) tree inferred from 18S rDNA sequences, showing the phylogenetic position of *Metagonostomoides elongatus* sp. nov. and *Wallackia koreana* sp. nov. (in bold red). GenBank accession numbers follow species names. Numbers at the nodes represent the ML bootstrap values/Bayesian inference (BI) posterior probabilities. Asterisks (*) indicate different topologies between BI and ML analyses. The sequences in the compressed clades are listed in Supplementary table S1. The scale bar corresponds to one substitution per 100 nucleotide positions

**Fig. 10:**
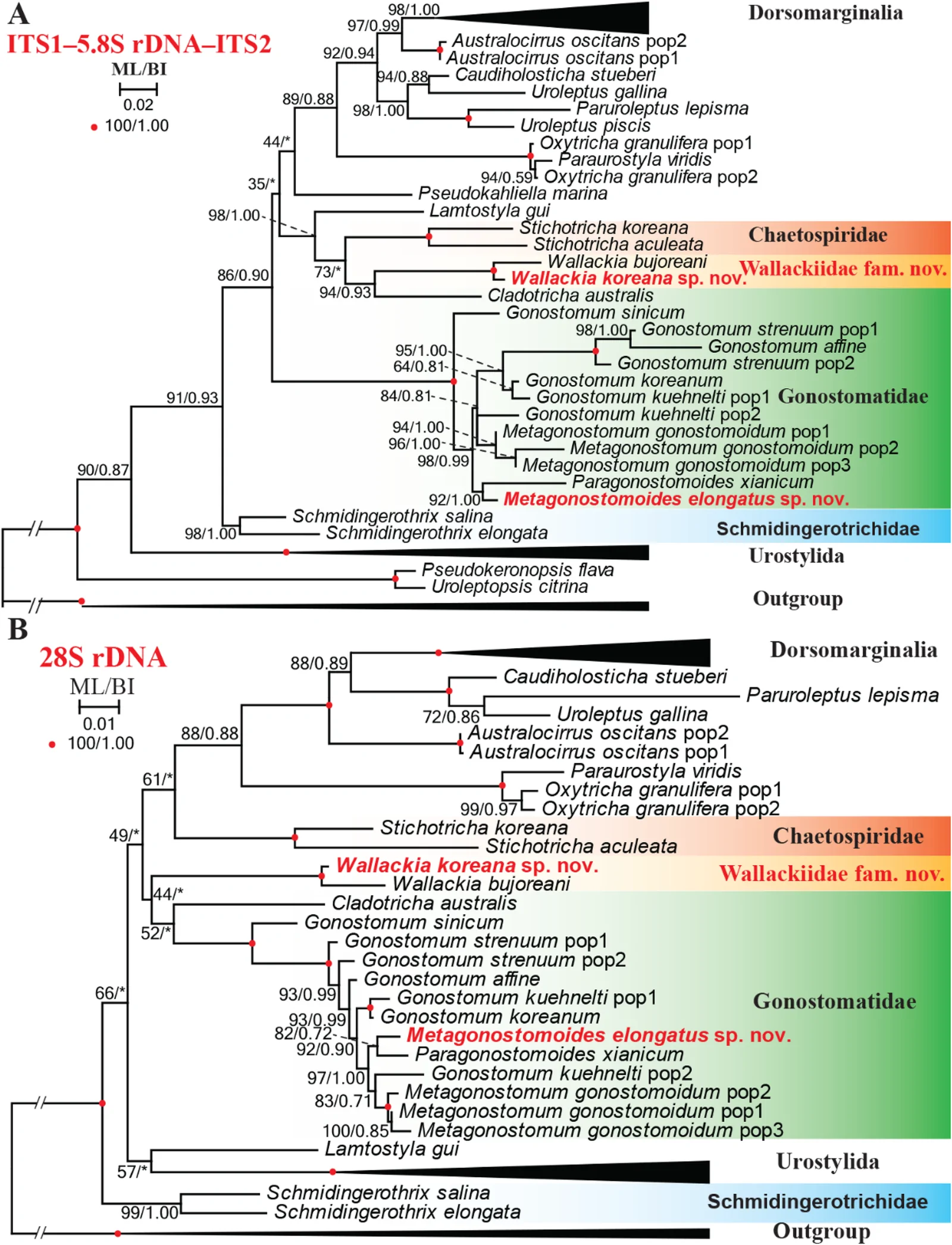
Maximum likelihood (ML) trees inferred from ITS1–5.8S rDNA–ITS2 (**A**) and 28S rDNA (**B**) sequences, showing the phylogenetic position of *Metagonostomoides elongatus* sp. nov. and *Wallackia koreana* sp. nov. (in bold red). Accession numbers are listed in Supplementary Table S2. Numbers at the nodes represent the ML bootstrap values and the Bayesian inference (BI) posterior probabilities. Asterisk (*) indicates different topologies between BI and ML analyses. The scale bars correspond to two (**A**) and one (**B**) substitution per 100 nucleotides

**Fig. 11:**
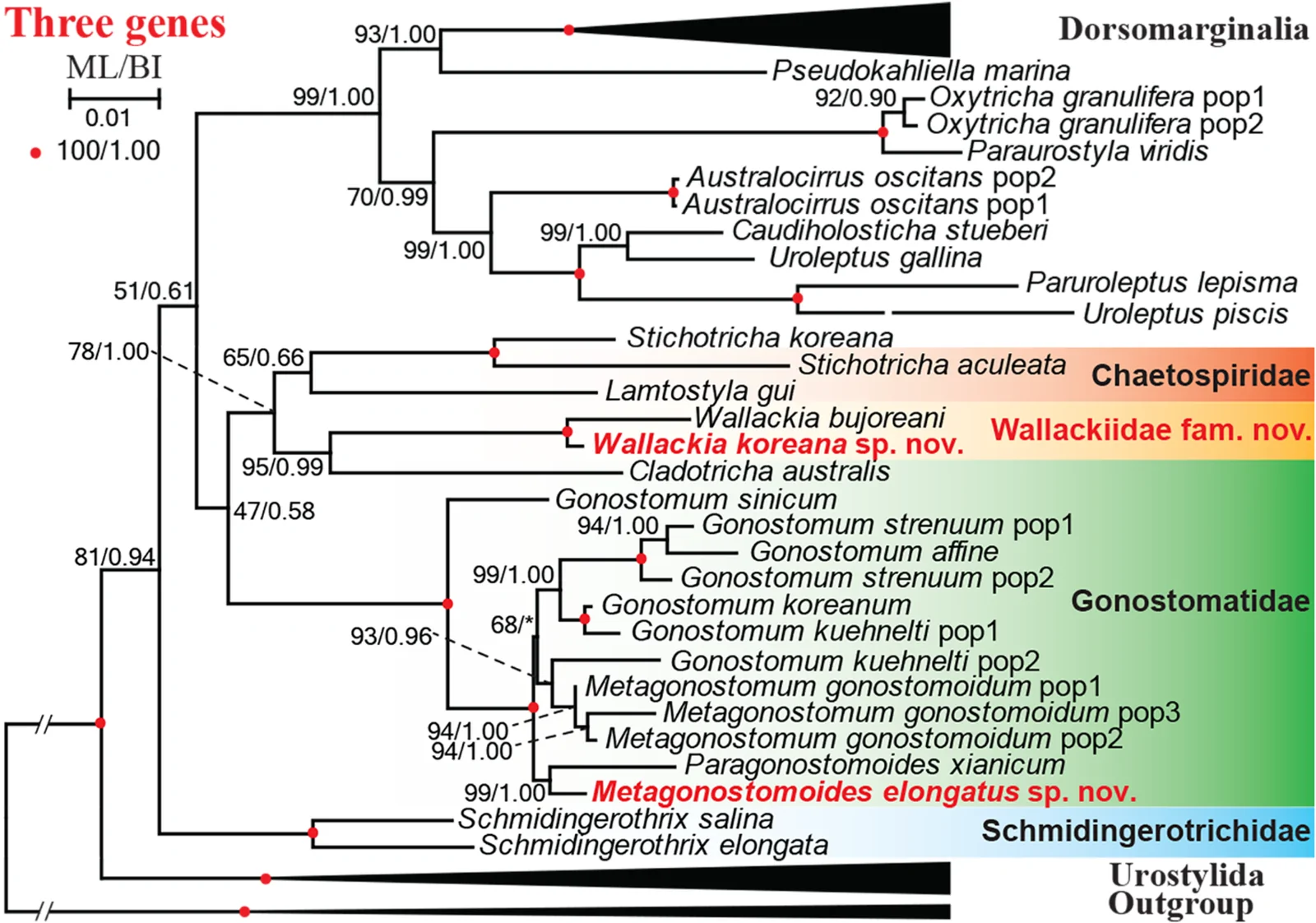
Maximum likelihood (ML) tree inferred from the multi-gene 18S–ITS1–5.8S–ITS2–28S rDNA sequences, showing the phylogenetic position of *Metagonostomoides elongatus* sp. nov. and *Wallackia koreana *sp. nov. (in bold red). Accession numbers are listed in Supplementary Table S2. Numbers at the nodes represent the ML bootstrap values and the Bayesian inference (BI) posterior probabilities. Asterisk (*) indicates different topologies between BI and ML analyses. The scale bar corresponds to one substitution per 100 nucleotides

The 18S–ITS1–5.8S–ITS2–28S rDNA sequences obtained from *Metagonostomoides elongatus* sp. nov. and *Wallackia koreana* sp. nov. were deposited in GenBank under accession numbers PZ307437 and PZ307438, respectively. The sequences are 5295 and 5296 bp long, with GC contents of 47.49% and 48.75%, respectively. Maximum-likelihood (ML) and Bayesian inference (BI) analyses produced broadly congruent topologies across datasets; therefore, we present only the ML tree for each marker/dataset, with ML bootstrap and BI posterior probability values shown at the nodes (Figs. 9, 10 and 11).

In the 18S rDNA phylogeny, *Metagonostomoides elongatus* sp. nov. clusters with three sequences of *Metagonostomum gonostomoidum* from China and Korea, with moderate ML support (80%) but low BI support (0.69). This assemblage forms a polytomy with *Paragonostomoides xianicum* and a clade comprising *Gonostomum koreanum* Omar et al., 2021 (MW471094) and *G. kuehnelti* Foissner, 1987 (MF445660, MW471095), supported by moderate ML (92%) but low BI (0.63). Other sequences identified as *Gonostomum* are distributed across several subclades, indicating non-monophyly of the genus in the 18S dataset (Fig. 9). The 18S rDNA sequence of *Wallackia koreana* sp. nov. groups with the only available sequence of *W. bujoreani* (Lepsi, 1951) Berger & Foissner, 1989 (MT247901) with maximal support (100% ML, 1.00 BI). Together, these two *Wallackia* sequences form a lineage near the base of a broader clade comprising representatives of Gonostomatidae, Chaetospiridae Jankowski in Small & Lynn, 1985, Schmidingerotrichidae Foissner, 2012, and Urostylida Jankowski, 1979, with moderate ML (75%) and low BI support (0.84).

In contrast to the 18S rDNA result, phylogenies inferred from ITS1–5.8S–ITS2, 28S rDNA, and the multi-gene rDNA dataset consistently place *Metagonostomoides elongatus* sp. nov. with *Paragonostomoides xianicum*. Support for this sister-group relationship is moderate to high across datasets (ITS1–5.8S rDNA–ITS2: 92% ML, 1.00 BI; 28S: 82% ML, 0.72 BI; multi-gene: 99% ML, 1.00 BI) (Figs. 10A and B and 11). In the ITS1–5.8S rDNA–ITS2 and multi-gene trees, the *M. elongatus* + *P. xianicum* clade branches basally to a subclade containing *Metagonostomum* and most available *Gonostomum* sequences, with *G. sinicum* Lu et al., 2017 excluded from that assemblage (Figs. 10A and 11). In the 28S rDNA tree, the *M. elongatus* + *P. xianicum* clade is recovered with a subclade comprising *Metagonostomum* sequences and a single sequence identified as *G. kuehnelti* (Fig. 10B).

Across all datasets, *Wallackia koreana* sp. nov. and *W. bujoreani* form a fully supported clade (100% ML, 1.00 BI) (Figs. 9, 10 and 11). The two species share sequence similarities of 99.1% (15 nucleotide differences) in the 18S rDNA gene, 96.5% (18 nucleotide differences) in the ITS1–5.8S rDNA–ITS2 region, and 98.5% (27 nucleotide differences) in the 28S rDNA gene. In the ITS1–5.8S rDNA–ITS2 and multi-gene trees, this *Wallackia* clade groups with *Cladotricha australis* within a subclade that also includes *Lamtostyla gui* and two *Stichotricha* sequences (Figs. 10A and 11). In the 28S rDNA tree, the two *Wallackia* sequences occur near the base of the clade comprising the available gonostomatid sequences, but this placement lacks statistical support (Fig. 10B).

## Discussion

### Comparison of *Metagonostomoides elongatus* sp. nov. with the most similar species

The new species is comparable only with gonostomatid taxa that possess long frontoventral cirral rows. Such forms are currently assigned to several genera, including *Gonostomum* (*G. koreanum*, *G. sinicum*, and *G. strenuum* (Engelmann, 1862) Sterki, 1878), *Metagonostomum gonostomoidum*, and *Paragonostomoides* Foissner, 2016 (*P. minutus* (Kamra et al., 2008) Foissner, 2016 and *P. xianicum*) [1, 5, 7, 11, 12, 17, 22, 23].

Morphologically, *M. elongatus* sp. nov. agrees with the three aforementioned *Gonostomum* species in having transverse cirri (distinctly separated from the frontoventral cirri) and caudal cirri. It can, however, be distinguished from all of them by the number and origin of its frontoventral cirral rows: in *M. elongatus* sp. nov. there are consistently five frontoventral cirral rows, whereas both *G. koreanum* and *G. strenuum* have six rows, and *G. sinicum* has either five or six rows [1, 5, 7, 11]. Ontogenetically, the new species differs from all gonostomatid taxa for which developmental data are available by the unique origin of the rightmost frontoventral (frontoterminal) cirral row: this row is formed from a combination of the two rightmost frontoventral anlagen, with its anterior portion derived from anlage V and its posterior portion from anlage IV. By contrast, in *Gonostomum* species with long frontoventral cirral rows, the frontoterminal row originates either de novo (*G. koreanum*: [1]; Australian population of *G. strenuum*: [3]), from both the oral primordium and the posterior cirri of the rightmost cirral row (*G. sinicum*: [11]), or solely from the oral primordium (Chinese population of *G*. *strenuum*: [7]). As already suggested by Omar et al. [1], this variability in the ontogenetic origin of the frontoterminal row among species currently assigned to *Gonostomum* is very likely a major factor underlying the non-monophyly of the genus in all present and previous molecular trees. Unfortunately, the lack of detailed morphological, ontogenetic, and molecular data for the type species, *G. affine* (Stein, 1859) Sterki, 1878, presently prevents a taxonomically sound resolution of this problem.

*Metagonostomoides elongatus* sp. nov. is also superficially similar to *Metagonostomum gonostomoidum*. The latter possesses six frontoventral cirral rows, of which only the rightmost (frontoterminal) cirral row is elongated and terminates near the transverse cirri, whereas each of the remaining cirral rows consists of only one or two cirri [1, 16, 17, 22, 23]. By contrast, the new species has invariably five frontoventral cirral rows, three of which are elongated and terminate anterior to mid-body, with the shortest (row III) comprising three or four cirri. Ontogenetically, *Metagonostomum gonostomoidum* can be clearly separated from *Metagonostomoides elongatus* sp. nov. by the origin of its frontoterminal row: in *Metagonostomum gonostomoidum* it arises intrakinetally at a single level within the parental row and then splits in the middle, as demonstrated by Hemberger [23] and Omar et al. [1], whereas in *Metagonostomoides elongatus* sp. nov. it is the product of two anlagen and thus of mixed origin.

*Paragonostomoides xianicum* is superficially similar to *M. elongatus* sp. nov. in having five frontoventral cirral rows, three of which are elongated, and in possessing a short row III with at least three cirri [12]. Furthermore, phylogenetic analyses based on the ITS1–5.8S–ITS2, 28S, and multi-gene datasets consistently place *M. elongatus* sp. nov. close to *P. xianicum*, suggesting a close evolutionary relationship between the two taxa. However, we do not consider *P. xianicum* to belong to *Metagonostomoides*. The strongest evidence comes from morphogenesis. In *P. xianicum*, primordium V develops intrakinetally within row V at two different levels, whereas in *M. elongatus* sp. nov., primordium V originates from both rows IV and V. This fundamental difference in the origin and development of primordium V represents a stable and important diagnostic character at the generic level. In addition, *P. xianicum* was originally described as lacking transverse cirri. Although the posteriormost cirrus of row V in the type population drawing appears distinctly displaced posteriorly and larger than the remaining cirri, suggesting that it may represent a transverse cirrus, this does not alter our taxonomic conclusion. Even if future observations confirm the presence of a transverse cirrus in *P. xianicum*, the distinct morphogenetic pattern still clearly supports that *P. xianicum* and *M. elongatus* sp. nov. belong to different genera.

*Paragonostomoides minutus* was only briefly described from its type locality in India. It can be easily distinguished from *Metagonostomoides elongatus* sp. nov. by having seven or eight macronuclear nodules (vs. two), a reduced number of frontoventral cirri (vs. frontoventral cirri arranged in rows each with several cirri), a posteriorly shortened left marginal row (vs. left marginal row ends terminally), and a paroral membrane reportedly composed of only two kinetosomes (vs. 9–14 kinetosomes) [17]. Because *P. minutus* is the type species of *Paragonostomoides*, comprehensive ontogenetic data, ideally complemented by molecular sequences, are essential to refine the generic diagnosis and to evaluate its relationship to *P. xianicum* and *Metagonostomoides* gen. nov.

*Neowallackia petergofi* (Alekperov, 2005) Berger, 2011 shows a comparable number and length of frontoventral cirral rows to *Metagonostomoides elongatus* sp. nov.; however, the presence of a single buccal cirrus suggests closer affinity with Gonostomatidae than with Wallackiidae fam. nov., in which a buccal cirral row is typical. Conversely, the absence of both transverse and caudal cirri excludes *N. petergofi* from *Metagonostomoides*, *Metagonostomum*, and *Paragonostomoides* [5, 24]. This combination of characters is more consistent with *Gonostomoides* Foissner, 2016, which likewise lacks transverse and caudal cirri, and *N. petergofi* may therefore be more appropriately placed in that genus pending detailed morphogenetic and molecular reassessment.

Taken together, *M. elongatus* sp. nov. differs from all morphologically similar gonostomatid species by a distinctive combination of frontoventral-row configuration, presence and arrangement of transverse cirri, and, most importantly, by its unique origin of the frontoterminal row from two separate anlagen, supporting its placement in a separate genus, *Metagonostomoides* gen. nov.

### Establishment of the new genus *Metagonostomoides* gen. nov.

The family Gonostomatidae comprises several genera that are, in most cases, delimited mainly by gross morphological characters [4, 5]. This situation reflects both the scarcity of detailed morphogenetic data for many species and the presence of incomplete or partly conflicting developmental accounts for others. A well-known example is *Gonostomum strenuum*: the Australian population studied by Foissner et al. [3] and the Chinese population investigated by Song [7] differ in the reported origin of the frontoterminal row, which arises de novo in the former but from the oral primordium in the latter. Such inconsistencies, together with the limited availability of molecular data for many nominal taxa, have contributed to taxonomic uncertainty and have sometimes led substantial developmental differences to be interpreted as intraspecific variation rather than as potential indicators of generic divergence.

In the present study, we discovered a new species that is morphologically most similar to members of *Metagonostomum* and *Paragonostomoides* and, more distantly, to some *Gonostomum* species. Because ontogenetic and molecular data are available for the relevant comparative taxa, the new species can be evaluated using an integrative framework. Although several *Gonostomum* species share aspects of the cirral pattern with the new taxon, none shows the distinctive morphogenetic feature observed here, namely, that the rightmost cirral row originates predominantly from anlage V with an additional short posterior contribution from anlage IV (i.e., pseudorow). This mixed contribution has not been reported from any *Gonostomum* species with known morphogenesis, supporting separation of the new taxon from *Gonostomum* at the generic level.

*Metagonostomum* (as currently understood and based on *M. gonostomoidum*) is characterized by six FVT cirral rows [1, 23]. In contrast, the new species consistently has five FVT rows and differs not only numerically but also developmentally: the rightmost row derives mainly from anlage V and incorporates a short posterior portion of anlage IV. These characters exclude the new species from *Metagonostomum*.

In the diagnosis of *Paragonostomoides* by Foissner [4], several characters relevant to the present comparison (e.g., the number of FVT rows and the condition of transverse cirri) are not emphasized. *Paragonostomoides xianicum* was assigned to the genus without an accompanying revision of the generic diagnosis and without ontogenetic data from the type species being available for direct comparison [12]. Pending a comprehensive re-evaluation of *Paragonostomoides* that includes morphogenesis of the type species, *Metagonostomoides elongatus* sp. nov. is readily distinguished from *P. minutus* and *P. xianicum* by the presence (vs. absence) of one or two transverse cirri and by its distinctive morphogenesis, in particular the predominant origin of the rightmost cirral row from anlage V with an additional posterior contribution from anlage IV [4, 12, 17].

Phylogenetic analyses provide an additional, but marker-dependent, perspective: the new species clusters with *Metagonostomum gonostomoidum* in the 18S rDNA tree, whereas ITS1–5.8S rDNA–ITS2, 28S rDNA, and multi-gene datasets place it with *Paragonostomoides xianicum* (Figs. 9, 10A and B and 11). This discordance indicates that rDNA markers alone do not permit a confident assignment of the species to either *Metagonostomum* or *Paragonostomoides*. When considered together with the diagnostic morphology and the unique morphogenetic pattern, the evidence supports recognition of an independent lineage, for which we establish *Metagonostomoides* gen. nov.

The genera *Wallackia* and *Neowallackia* also possess long cirral rows but are readily distinguished from *Metagonostomoides* gen. nov. by having at least two buccal cirri (vs. a single buccal cirrus), lacking transverse cirri (vs. present), and exhibiting independent formation of proter and opisthe FVT anlagen (vs. shared primary primordia). In addition, *Neowallackia* differs from *Metagonostomoides* gen. nov. by the absence (vs. presence) of caudal cirri [3, 5, 25, 26].

### Comparison of *Wallackia koreana* sp. nov. with the congeners

Members of *Wallackia* and *Neowallackia* share a gonostomatid-type oral apparatus and long frontoventral cirral rows (including a buccal cirral row). The two genera are most readily distinguished by the presence (in *Wallackia*) vs. absence (in *Neowallackia*) of caudal cirri; accordingly, the new species is assigned to *Wallackia*. At present, three species are recognized in the genus: *W. schiffmanni* Foissner, 1976 (type species), *W. bujoreani*, and *W. elegans* Foissner et al., 2002 [3, 5, 26, 27].

*Wallackia schiffmanni* is difficult to distinguish from the Namibian population identified as *W. bujoreani* by Foissner et al. [3], as both exhibit similar numbers of cirri in the buccal row and in frontoventral cirral row III. The two taxa differ mainly in the spacing of the caudal cirri (widely vs. narrowly separated) and in the presence vs. absence of elongated dorsal bristles at the posterior end of the body [3, 26]. *Wallackia koreana* sp. nov. likewise differs from *W. schiffmanni* in these two characters and can be further separated by its smaller body size (50–70 × 15–23 μm vs. 85–100 × 25–33 μm), and by having fewer cirri in both the buccal row (2 vs. 6) and frontoventral cirral row III (3–4 vs. 6) [26].

Foissner et al. [3] reported two populations of *Wallackia elegans* from Namibia and Tenerife (Canary Islands). *Wallackia elegans* is characterized by its small size and a lanceolate body with a narrowed posterior end, typically curved rightward. *Wallackia koreana* sp. nov. is comparable in body size to *W. elegans* (50–70 × 15–23 μm vs. 50–70 × 15–20 μm in vivo) but differs clearly in body shape, being ellipsoidal rather than lanceolate with a narrowed, right-curved posterior end. Moreover, *W. koreana* sp. nov. has more adoral membranelles (23–29 vs. 16–20), fewer buccal cirri (2 vs. 2–5), more cirri in frontoventral cirral row IV (11–16 vs. 5–7), fewer cirri in row V (11–17 vs. 12–22), and distinctly shorter caudal cirri (about 15 μm vs. about 25 μm in vivo).

Since its original description, *W. bujoreani* has been reported repeatedly, but published accounts differ markedly in cirral counts. Lepsi [25], based solely on live observations of the Romanian type population, illustrated a buccal cirral row comprising ~ 12 cirri and an adjacent frontoventral cirral row (III) with 15 cirri. By contrast, Berger & Foissner [27], in their neotypification of the species, described a French population with only two or three buccal cirri and only two to four cirri in frontoventral cirral row III. Likewise, the Namibian population reported by Foissner et al. [3] differs again, with four buccal cirri in the life illustration of morphostatic specimen but six in all dividing specimens, and with frontoventral cirral row III comprising four cirri in the morphostatic specimen and four or five in dividing specimens. Notably, the protargol-impregnated specimen provided by Foissner et al. [3] (Fig. 398 l) shows only two buccal cirri, raising the possibility that the Namibian material contained more than one species. Taken together, these discrepancies suggest that populations identified as *W. bujoreani* may not be conspecific and that the taxonomy of this species requires re-evaluation.

Compared with the reported populations of *W. bujoreani*, *Wallackia koreana* sp. nov. is clearly distinct from the Romanian type population [25] by its much lower number of buccal cirri (2 vs. ~12) and by the markedly reduced cirral number in frontoventral cirral row III (3–4 vs. ~15) [25]. It also differs from the Namibian population by having fewer buccal cirri (2 vs. 4–6) [3]. Although the specimen illustrated as Fig. 398 l in Foissner et al. [3] is strikingly similar to the new species, it contrasts strongly with the remaining illustrated specimens of the Namibian population, suggesting that it may represent an accidentally included individual from a different population (or even a different species) rather than *W. bujoreani* sensu Foissner et al. [3]. *Wallackia koreana* sp. nov. is most similar to the French neotype population of *W. bujoreani* investigated by Berger & Foissner [27], sharing comparable counts of adoral membranelles and cirri in the buccal row and frontoventral cirral rows. Nevertheless, the new species differs by its smaller size after protargol impregnation (42–57 × 12–18 μm vs. 52–80 × 17–36 μm) and by a relatively longer adoral zone of membranelles (54–72% vs. ~50% of body length). Given the pronounced variability and potential taxonomic inconsistency among populations assigned to *W. bujoreani*, we cannot exclude that the French population represents *W. koreana* sp. nov.; however, this hypothesis requires confirmation with molecular data. At present, the only sequence deposited in GenBank under the name *W. bujoreani* is not linked to a documented morphology. Its consistent clustering with *W. koreana* sp. nov. across all analyses (Figs. 9, 10 and 11) supports placement within *Wallackia*, but a precise species assignment remains uncertain until voucher morphology is available for that sequenced population [6].

### Reassessment of gonostomatid morphogenesis reveals family-level divergence and supports Wallackiidae fam. nov

In hypotrich ciliates, ontogenetic traits are often more informative than gross morphology for delimiting higher-level taxa. A classic example is dorsal kinety fragmentation, which is the key apomorphy defining the families Oxytrichidae Ehrenberg, 1830, Neokeronopsidae Foissner & Stoeck, 2008, and Spirofilidae Gelei, 1929 [28–37]. By contrast, patterns in the development of the FVT cirral apparatus have received comparatively little attention, likely because detailed ontogenetic data are still sparse for many hypotrich families.

In most members of Gonostomatidae, the frontoventral anlagen arise as shared primary primordia (typically anlagen II–VI, or a subset thereof) common to both proter and opisthe; these primordia then divide transversely to produce the secondary primordia allocated to each filial cell [1, 5, 7–9, 11, 12]. A comparable arrangement is also characteristic of Amphisiellidae [38–43]. In contrast, most other hypotrich lineages follow different developmental schemes: in Urostylida, Uroleptidae Foissner & Stoeck, 2008, Neokeronopsidae, Spirofilidae, and most Stylonychinae Berger & Foissner, 1997, the opisthe FVT anlagen arise independently from the outset, whereas in most non-stylonychine oxytrichids and in non-oxytrichid Dorsomarginalia (taxa with an oxytrichid-like FVT pattern but lacking dorsal kinety fragmentation) only anlagen V and VI are initially shared and are subdivided later [28, 30, 34–37, 43–45].

*Metagonostomoides* gen. nov. agrees with *Gonostomum*, *Metagonostomum*, and *Paragonostomoides*, i.e., the genera forming the core Gonostomatidae clade, in having frontoventral cirral rows that originate as common primary primordia for proter and opisthe [1, 5, 12]. This supports treating these genera as members of a single family and indicates that the presence of shared primary primordia is a family-level character.

Within this framework, *Cotterillia bromelicola* Foissner & Stoeck, 2011 is a striking outlier. In both our and previous 18S rDNA trees, *Cotterillia* consistently clusters within the *Gonostomum* clade and appears as a long, isolated branch. However, this topological position should not be interpreted as evidence of close generic affinity. *Cotterillia* is already recognized as a distinct genus and differs profoundly from *Gonostomum* in both morphology and ontogenesis. It possesses cirral rows on both the ventral and dorsal sides of the body, and its dorsal kineties arise de novo, whereas other gonostomatids have three intrakinetally developing dorsal kineties. Furthermore, in *Cotterillia* the frontoventral anlagen of proter and opisthe (except anlage II) are formed independently; in all other genera of the core Gonostomatidae clade (*Gonostomum*, *Metagonostomoides* gen. nov., *Metagonostomum*, and *Paragonostomoides*), almost all frontoventral anlagen originate as common primary primordia that subsequently split between the two daughter cells. Taken together, these differences indicate that *Cotterillia* represents a lineage that is morphologically and developmentally far removed from *Gonostomum*, and its placement among *Gonostomum* sequences in a single-gene tree is most plausibly a long-branch or marker-choice artefact rather than a reflection of true generic relationships. The unique combination of a gonostomatid-like body and oral apparatus with a highly divergent dorsal pattern and divisional mode suggests that *Cotterillia* may ultimately warrant recognition of its own family-level group. For now, however, we regard any formal family proposal as premature until additional species and multigene datasets become available [46].

The two tailed gonostomatids, *Apogonostomum pantanalense* Foissner, 2016 and *Gonostomum namibiense* Foissner et al., 2002, consistently fall outside the core Gonostomatidae clade in the 18S rDNA tree. Both differ markedly from the core gonostomatid genera in overall body morphology, particularly in having a distinctly tailed posterior end rather than the blunt posterior typical of all species in the core clade. They also possess rod-shaped cortical granules (extrusomes) arranged as a subcortical seam. In addition, *G. namibiense* exhibits an unstable frontoventral cirral pattern, whereas *A. pantanalense* possesses ventral cirral pairs extending almost to the transverse cirri [3, 4]. Taken together, the available morphological and molecular evidence suggests that posterior body shape (tailed vs. blunt) may represent an important higher-level character within the broader gonostomatid assemblage. The presence of distinctive rod-shaped cortical granules further supports the separation of these tailed taxa from core members of the Gonostomatidae as suggested by Fan et al. [47]. However, because ontogenetic data and molecular markers remain unavailable for many tailed taxa, including members of *Paragonostomum* Foissner et al., 2002, *Gonostomoides caudatus* Foissner, 2016, and *Gonostomum caudatum* Foissner, 2016, formal taxonomic conclusions should be deferred until more comprehensive data become available [4].

Within hypotrichs bearing a gonostomatid-type oral apparatus, *Wallackia* and *Neowallackia* clearly deviate from the “typical” gonostomatid pattern in both morphology and morphogenesis. Both genera possess a buccal cirral row rather than the single buccal cirrus characteristic of gonostomatid genera (with few exceptions in *Cladotricha*) [3, 26, 27, 48]. Morphogenetically, they lack shared primary frontoventral primordia: the proter and opisthe FVT anlagen arise independently from the outset, whereas in core gonostomatids they originate as common (shared) primordia that subsequently split between the two daughter cells [3, 48]. Consistent with these morphological and ontogenetic differences, the 18S rDNA sequences of *Wallackia koreana* sp. nov. and *W. bujoreani* are recovered near the base of a clade comprising Chaetospiridae, Schmidingerotrichidae, and Urostylida (Fig. 9), while the ITS1–5.8S rDNA–ITS2 and multi-gene rDNA analyses place them with Chaetospiridae (Figs. 10A and 11). This interpretation is further supported by their affinity to Chaetospiridae, which likewise combines a gonostomatid-type oral apparatus with long frontoventral cirral rows, although these rows are straight in *Wallackia* and *Neowallackia* rather than spiraled as in chaetospirids. In addition, most species of *Wallackia* and *Neowallackia* possess rod-shaped cortical granules (extrusomes), a feature recently reassessed by Fan et al. [47] as potentially representing a high-rank taxonomic character. However, because this character is absent in at least *Neowallackia franzi* (Foissner, 1982) Berger, 2011, it cannot presently be regarded as a diagnostic feature of the group. Nevertheless, its frequent occurrence provides additional support for the distinctiveness of this lineage. Taken together, the distinct divisional mode, buccal cirral organization, phylogenetic position, and frequent occurrence of rod-shaped cortical granules indicate that *Wallackia* and *Neowallackia* are not members of Gonostomatidae, but instead represent a separate lineage warranting family rank, Wallackiidae fam. nov.

Members of the genus *Cladotricha* Gaievskaïa, 1925 also fall outside the core Gonostomatidae clade in all phylogenetic trees, usually near *Wallackia*, which is consistent with their close morphological similarity. Small and Lynn [49] originally established the family Cladotrichidae for *Cladotricha*, but Berger [5] later synonymized Cladotrichidae with Gonostomatidae, mainly because *Cladotricha* possesses a gonostomatid-type oral apparatus and lacks dorsal kinety fragmentation. However, *Cladotricha* is morphologically and ontogenetically heterogeneous. The number of buccal cirri, for example, varies markedly among species: it is absent in *C. koltzowii* Gaievskaïa, 1925, represented by a single cirrus in *C. halophila* Wilbert, 1995 and *C. sagittata* Ruinen, 1938, and comprises two or more cirri in *C. australis* Blatterer & Foissner, 1988, *C. niesseniae* Li et al., 2020, and *C. sigmoida* Ruinen, 1938 [5, 50–53]. Ontogenetic data further show that the frontoventral anlagen originate independently (rather than as shared primary primordia) in some species, such as *C. koltzowii* and *C. halophila*. Notably, *C. niesseniae*, described by Li et al. [52] as conspecific with the population characterized by Nießen [54], displays a different morphogenetic pattern, namely an independent (vs. shared) origin of frontoventral anlage IV in proter and opisthe. This suggests that, despite their similar morphology, the two populations likely represent distinct species. Taken together, the inconsistency in buccal cirri number, the variability in FVT anlagen origin, and the lack of comprehensive morphological and molecular data for the type species make the status of *Cladotricha* and Cladotrichidae difficult to resolve at present. We therefore provisionally follow Berger [5] in synonymizing Cladotrichidae with Gonostomatidae, while emphasizing that a thorough redescription of the type species and broader taxon sampling will be essential to test this arrangement.

### Reassessment of the “gonostomatid-type” oral apparatus across hypotrichs

According to Berger [5], the main apomorphy of gonostomatids is their conspicuous oral apparatus: the adoral zone of membranelles (AZM) runs mainly along the left body margin, with its proximal portion bending towards the cell midline to form a characteristic “knee-shaped” curve; the paroral membrane consists of widely spaced kinetids, whereas the endoral is composed of densely packed kinetids.

Within Gonostomatidae, *Afrogonostomum alveum* Foissner & Berger, 2021 was diagnosed as having a paroral membrane composed of dikinetids. Under Berger’s [5] concept of the gonostomatid oral apparatus, and consistent with our interpretation, this condition departs from the typical gonostomatid pattern and suggests that *A. alveum* may not belong in Gonostomatidae. Moreover, its frontoventral cirral arrangement, the presence of transverse cirri, and the absence of caudal cirri closely match the condition in *Parasincirra* Ma et al., 2021. The main discrepancy is the presence vs. absence of a buccal cirrus. Taken together, these characters indicate that *Afrogonostomum* Foissner & Berger, 2021 is unlikely to be a gonostomatid and may instead be more closely related to *Parasincirra*, a hypothesis that should be tested with detailed morphogenetic data and molecular sequences [21, 55].

In the family Schmidingerotrichidae, the paroral is entirely absent, so the oral apparatus departs fundamentally from the gonostomatid pattern; this configuration is best treated as a separate *Schmidingerothrix*-type [56–59]. Members of *Urosoma* Kowalewski, 1882 have previously been described as possessing a gonostomatid-like oral apparatus, but a careful re-examination of the original descriptions and illustrations suggests otherwise. In *Urosoma*, the AZM does not form a clear knee-shaped bend; in some species the endoral is composed of dikinetids; and in several cases the paroral kinetids appear closely apposed rather than widely spaced. Accordingly, the oral apparatus of *Urosoma* should not be regarded as genuinely gonostomatid. Specifically, it differs both in the overall shape of the AZM and in the highly variable arrangement of kinetids in the paroral, which has been described as consisting of dikinetids, densely spaced kinetids, or widely spaced monokinetids. At least some of this variation may reflect misinterpretations or inaccuracies in the descriptions and drawings [27, 60–66].

Similarly, members of the family Kahliellidae Tuffrau, 1979 have often been treated as possessing a gonostomatid-type oral apparatus, but a closer examination indicates otherwise. Their AZM closely resembles that of *Urosoma*, and the paroral consists of a row of monokinetids, as in *Perisincirra* Jankowski, 1978, or they exhibit an AZM similar to that of *Gonostomum* but with a paroral composed of a row of dikinetids, as in *Kahliella matisi* Vďačný et al., 2010 [67, 68]. Likewise, in species such as *Pseudokahliella marina* (Foissner et al., 1982) Berger et al., 1985, Wang et al. [6] interpreted the oral apparatus as “*Gonostomum*-type”, but both paroral and endoral are composed of closely arranged, densely packed kinetids, which does not match the combination of a widely spaced paroral and densely spaced endoral that typifies gonostomatid taxa [69, 70].

Taken together, these observations indicate that several lineages traditionally claimed to have “gonostomatid-like” oral structures actually deviate in key respects from Berger’s [5] concept. If his defining criteria are applied strictly, only a limited set of lineages can be regarded as bearing a true “gonostomatid-type” oral apparatus, namely the families Gonostomatidae, Chaetospiridae, and Wallackiidae fam. nov.

## Conclusions

This study shows that combining morphology, morphogenesis, and multigene phylogenetics is essential for resolving relationships among hypotrichs with a gonostomatid oral apparatus. We describe two new soil species from Korea: *Metagonostomoides elongatus* gen. nov., sp. nov., distinguished by a unique FVT divisional pattern and a marker-dependent phylogenetic position between *Metagonostomum* and *Paragonostomoides*, and *Wallackia koreana* sp. nov., expanding the known diversity of *Wallackia*. A family-wide review indicates that shared primary primordia are conserved in core Gonostomatidae, whereas *Wallackia* and *Neowallackia* form proter and opisthe anlagen independently, supporting Wallackiidae fam. nov. Re-evaluation of the gonostomatid-type oral apparatus restricts it to Gonostomatidae, Chaetospiridae, and Wallackiidae fam. nov.

## Methods

### Sample collection, cultivation, preparation, and identification

*Metagonostomoides elongatus* gen. nov., sp. nov. was discovered in a soil sample collected from Cheongdae Mountain, Sokcho-si, Gangwon-do, South Korea, on 19 July 2019. The sample was air-dried for two weeks, stored in a plastic bag, and rewetted in December 2020 with mineral water (Jeju Samdasoo, Jeju Province Development Co., South Korea) to induce excystment using the non-flooded Petri dish method [71]. A clonal culture was established in a plant culture dish containing mineral water at room temperature; small pieces of mealworm were added to promote bacterial growth as a food source. *Wallackia koreana* sp. nov. was obtained from a water sample containing debris collected from a temporary puddle on a grass lawn at Kangwon National University, on 1st of July 2020. Attempts to establish a clonal culture were unsuccessful; only a limited number of cells were detected in a *Notohymena* sp. culture derived from the same sample.

Living specimens were investigated using a stereomicroscope (Olympus SZ61, Tokyo, Japan) and light microscope (Olympus BX53) with differential interference contrast at magnifications of 50–1,000×. The infraciliature was revealed by protargol impregnation. Protargol powder was synthesized using the method of Pan et al. [72] and Kim & Jung [73], and the protargol impregnation technique is based on ‘procedure A’ of Foissner [74]. Terminology is according to Berger [5], Foissner [4], and Omar et al. [1].

### DNA extraction, PCR amplification, and sequencing

Five cells from each species were collected using glass pipettes under the stereomicroscope. The cells were washed at least five times using sterile distilled water to remove yeast cells and other contaminants and then each cell was transferred to a 1.5 mL centrifuge tube with a minimum volume of water. Genomic DNA was extracted using a RED-Extract-N-Amp Tissue PCR Kit (Sigma, St. Louis, MO, USA). Two PCR reactions were conducted to amplify ribosomal genes as follows: (1) primers New Euk A and LSU rev4 were used to amplify 18S rRNA, ITS1, 5.8S rRNA, ITS2, and partial 28S rRNA genes [75, 76], under the conditions in Park et al. [77]; (2) primers 28S-1F and 28S-4R were used to amplify a longer fragment of the 28S rRNA, following the PCR conditions used by Moreira et al. [78]. PCR products were purified using the MEGAquickspin Total Fragment DNA Purification Kit (iNtRON, South Korea). DNA sequencing was performed bidirectionally on an ABI 3700 sequencer (Applied Biosystems, Foster City, CA, USA). For the first primer set, internal sequencing primers 18SR300, 18SF790v2, and 18SF1470 were used [77, 79]. For the second primer set, internal primers 28S-568F and 28S-2F were used [78]. Resulting sequence fragments from the two sets were assembled using Geneious 9.1.8 [80].

### Phylogenetic analyses

To determine the phylogenetic position of the two new species, four datasets were constructed according to Omar et al. [1] as follows: (i) 18S rDNA (consisting of 151 sequences; 1713 characters); (ii) ITS1–5.8S rRNA–ITS2 (74 sequences; 640 characters); (iii) partial 28S rDNA (73 sequences; 1843 characters); and (iv) three concatenated rDNA regions (74 sequences; 4293 characters). Three euplotids were used as outgroup taxa: *Apodiophrys ovalis*,* Diophrys scutum*, and *Paradiophrys zhangi*. The four datasets were aligned using ClustalW [81], and both ends of each alignment were trimmed in BioEdit 7.0.9.0 [82]. jModelTest 2.1.7 [83] was used to select the best-fit model GTR + I + G for the first, third, and fourth datasets and TIM2 + I + G for the second dataset under the Akaike information criterion (AIC). The maximum likelihood (ML) analyses were conducted using IQ-Tree 1.5.3 [84] with 100,000 ultrafast bootstrap replicates. MrBayes 3.2.6 [85] was used for Bayesian inference (BI) analyses with Markov chain Monte Carlo simulations running for 5,000,000 generations with a sampling frequency of every 100 generations until the mean standard deviation of the frequencies fell below 0.01. The first 25% of the trees were discarded as burn-in. Phylogenetic trees were visualized using MEGA 6.06 [86] and the free software package FigTree v1.4.3 (A. Rambaut; http://tree.bio.ed.ac.uk/software/figtree/).

The bootstrap values are interpreted following Hillis & Bull [87], that is, values ≥ 95% are considered high, from 71% to 94% as moderate, from 50% to 70% as low, and < 50% as without support. Bayesian posterior probability values are interpreted following Alfaro et al. [88], that is, ≥ 0.95 are considered high and < 0.95 as low.

## Supplementary Information

Supplementary Material 1. Supplementary Table S1 Taxon names and GenBank accession numbers in the compressed clades of the 18S rDNA phylogenetic tree in Fig. 9. Supplementary Table S2 List of taxa with GenBank accession numbers of corresponding 18S–ITS1–5.8S–ITS2–28S rDNA sequences included in second, third, and fourth datasets (Figs. 10 and 11).

## Data Availability

The datasets generated and/or analysed during the current study are available in the GenBank repository. The rDNA sequences have been deposited in GenBank under the accession numbers PZ307437 (*Metagonostomoides elongatus*) and PZ307438 (*Wallackia koreana*).
