# Supplementary material for: Revealing hidden diversity in gonostomatid ciliates (Ciliophora, Hypotrichia): integrative evidence supports two new species, a new genus, and Wallackiidae fam. nov
Source: BMC Microbiol. 2026 Jul 11;26:863. doi: 10.1186/s12866-026-05385-x (PMC13640345; doi:10.1186/s12866-026-05385-x)
Supplement: Supplementary file 1 [file 12866_2026_5385_MOESM1_ESM.docx]

**Supplementary Table S1** Taxon names and GenBank accession numbers in the compressed clades of the 18S rDNA phylogenetic tree in Fig. 9.

| Species | GenBank Accession No. |
| --- | --- |
| Dorsomarginalia | |
| *Afrokeronopsis aurea* | EU124669 |
| *Apoamphisiella vernalis* | KU522216 |
| *Aponotohymena apoaustralis* | KC430934 |
| *Aponotohymena australis* | MG994983 |
| *Aponotohymena isoaustralis* | KP336402 |
| *Apoterritricha lutea* | KJ619458 |
| *Australocirrus aspoecki* | MN562621 |
| *Australocirrus australis* | KP100452 |
| *Australocirrus australis* | MN559078 |
| *Cyrtohymena citrina* | KC182574 |
| *Cyrtohymena muscorum* | KM061384 |
| *Gastrostyla steinii* | AF508758 |
| *Hemiurosomoida longa* | JX893371 |
| *Hemiurosomoida tibetensis* | MZ457562 |
| *Histriculus histrio* | FM209294 |
| *Hypotrichidium conicum* | MW830115 |
| *Hypotrichidium paraconicum* | JQ918371 |
| *Hypotrichidium tisiae* | ON117316 |
| *Laurentiella strenua* | AJ310487 |
| *Metahymena inquieta* | KM923764 |
| *Metasterkiella koreana* | KY448243 |
| *Notohymena antarctica* | MZ710044 |
| *Notohymena gangwonensis* | MN977117 |
| *Onychodromopsis flexilis* | AM412764 |
| *Onychodromus grandis* | AJ310486 |
| *Paraparentocirrus sibillinensis* | KF184655 |
| *Parasterkiella thompsoni* | KM061387 |
| *Paraurostyla weissei* | AJ310485 |
| *Paraurostyla wuhanensis* | MN137908 |
| *Pattersoniella vitiphila* | JX885704 |
| *Pleurotricha lanceolata* | AF164128 |
| *Polystichothrix monilata* | KT192639 |
| *Pseudocyrtohymena koreana* | KX138654 |
| *Pseudocyrtohymenides lacunae* | MF319121 |
| *Pseudogastrostyla flava* | KP266627 |
| *Rigidohymena candens* | KC414885 |
| *Rigidohymena quadrinucleata* | KX925220 |
| *Rubrioxytricha ferruginea* | AF370027 |
| *Rubrioxytricha guamensis* | KY947508 |
| *Rubrioxytricha haematoplasma* | KJ645977 |
| *Rubrioxytricha tsinlingensis* | KR817675 |
| *Sterkiella cavicola* | GU942565 |
| *Sterkiella histriomuscorum* | FJ545743 |
| *Sterkiella nova* | AF508771 |
| *Sterkiella sinica* | KR817676 |
| *Sterkiella subtropica* | KM924307 |
| *Stylonychia ammermanni* | FM209295 |
| *Stylonychia lemnae* | AF508773 |
| *Stylonychia mytilus* | AF508774 |
| *Stylonychia nodulinucleata* | KY353799 |
| *Tetmemena pustulata* | AF508775 |
| Deviatidae | |
| *Deviata bacilliformis* | KJ766110 |
| *Deviata brasiliensis* | KP266620 |
| *Perisincirra paucicirrata* | JX012184 |
| Hemiholostichidae | |
| *Hemiholosticha kahli* | MK211833 |
| *Psilotrichides hawaiiensis* | MK211834 |
| *Urospinula succisa* | KF411460 |
| Strongylidiidae | |
| *Pseudouroleptus caudatus* | DQ910904 |
| *Strongylidium orientale* | KC153532 |
| *Strongylidium guangdongense* | MF113406 |
| Uroleptidae | |
| *Caudiholosticha stueberi* | KT724201 |
| *Paruroleptus lepisma* | AF164132 |
| *Uroleptus gallina* | AF164130 |
| *Uroleptus longicaudatus* | KF734979 |
| *Uroleptus piscis* | AF164131 |
| Urostylida | |
| *Anteholosticha antecirrata* | KU234525 |
| *Anteholosticha manca* | DQ503578 |
| *Anteholosticha paramanca* | KF806443 |
| *Apobakuella fusca* | JN008942 |
| *Apokeronopsis wrighti* | EU417963 |
| *Australothrix xianiensis* | KT892731 |
| *Bakuella granulifera* | KJ958489 |
| *Bakuella litoralis* | KR024010 |
| *Bakuella subtropica* | KC631826 |
| *Birojimia soyaensis* | MK959231 |
| *Diaxonella pseudorubra* | GU942564 |
| *Diaxonella trimarginata* | JQ424833 |
| *Metaurostylopsis alrasheidi* | MT911525 |
| *Metaurostylopsis cheni* | FJ775720 |
| *Neourostylopsis flava* | KR013238 |
| *Nothoholosticha flava* | KR612271 |
| *Pseudokeronopsis rubra* | HM140387 |
| *Thigmokeronopsis stoecki* | EU220226 |
| *Urostyla grandis* | AF164129 |
| *Urostyla grandis* | AF508781 |
| *Urostylida* sp. | KJ958488 |
| Arcuseriids | |
| *Amphisiella milnei* | FJ870072 |
| *Arcuseries scutellum* | FJ156105 |
| *Arcuseries petzi* | KM222093 |
| Parapirojimidae | |
| *Parabirojimia similis* | DQ503584 |
| *Tunicothrix brachysticha* | GU574811 |
| *Tunicothrix wilberti* | GU437210 |
| Outgroup | |
| *Apodiophrys ovalis* | GU477634 |
| *Diophrys scutum* | JF694040 |
| *Paradiophrys zhangi* | FJ870076 |

**Supplementary Table S2** List of taxa with GenBank accession numbers of corresponding 18S–ITS1–5.8S–ITS2–28S rDNA sequences included in second, third, and fourth datasets (Figs. 10 and 11).

|  | Species | GenBank accession numbers | | |
| --- | --- | --- | --- | --- |
|  |  | 18S | ITS1–5.8S–ITS2 | Partial 28S |
| 1 | *Afrokeronopsis aurea* | PV667846 | PV667835 | PV667855 |
| 2 | *Anteholosticha monilata* | KJ958488 | KJ958521 | |
| 3 | *Antetetmemena koreana* | PQ037621 | | |
| 4 | *Apoamphisiella tihanyiensis* | PV777671 | PV777678 | PV777683 |
| 5 | *Apodiophrys ovalis* | GU477634 | JF694069 | JF694045 |
| 6 | *Aponotohymena australis* | PV777670 | PV777677 | PV777682 |
| 7 | *Apostylonychia baugilensis* | PQ037619 | | |
| 8 | *Apostylonychia notophorides* | PQ037620 | | |
| 9 | *Australocirrus oscitans* pop1 | PV667848 | PV667837 | PV667857 |
| 10 | *Australocirrus oscitans* pop2 | PV667849 | PV667838 | PV667858 |
| 11 | *Australocirrus rubrus* pop1 | PV667850 | PV667841 | PV667861 |
| 12 | *Australocirrus rubrus* pop2 | PV667851 | PV667842 | PV667862 |
| 13 | *Australocirrus rubrus* pop3 | PV667852 | PV667843 | PV667863 |
| 14 | *Australocirrus shii* | PV667847 | PV667836 | PV667856 |
| 15 | *Bakuella granulifera* | KJ958489 | KJ958522 | |
| 16 | *Caudiholosticha stueberi* | PV667854 | PV667845 | PV667865 |
| 17 | *Cladotricha australis* | MT233385 | MT233381 | MT233384 |
| 18 | *Crassohymena primicirrata* | PP230160 | PV667839 | PV667859 |
| 19 | *Cyrtohymena citrina* | AF508755 | | |
| 20 | *Cyrtohymena muscorum* | PV777669 | PV777676 | PV777681 |
| 21 | *Cyrtohymena seorakensis* | MT103333 | PV667840 | PV667860 |
| 22 | *Diophrys scutum* | JF694040 | JF694073 | JF694054 |
| 23 | *Eschaneustyla lugeri* | KY874005 | KY874007 | KY874006 |
| 24 | *Extraholosticha sylvatica* | KJ958490 | KJ958523 | |
| 25 | *Gastrostyla steinii* | AF508758 | | |
| 26 | *Gonostomum affine* | KU594638 | KU594628 | KU594647 |
| 27 | *Gonostomum koreanum* | PZ307439 | | |
| 28 | *Gonostomum kuehnelti* pop1 | PZ307440 | | |
| 29 | *Gonostomum kuehnelti* pop2 | MT247885 | MT247888 | MT247892 |
| 30 | *Gonostomum sinicum* | KY475614 | MT233441 | MT233439 |
| 31 | *Gonostomum strenuum* pop1 | KU594639 | KU594629 | KU594648 |
| 32 | *Gonostomum strenuum* pop2 | MT247894 | MT247898 | MT247896 |
| 33 | *Hypotrichidium bichromum* | PV777673 | PV777680 | PV777685 |
| 34 | *Hypotrichidium paraconicum* | JQ918371 | KP266639 | KP266657 |
| 35 | *Lamtostyla gui* | MN733822 | | |
| 36 | *Laurentiella strenua* | PV781200 | PV781205 | PX789314 |
| 37 | ***Metagonostomoides elongatus* sp. nov.** | **PZ307437** | | |
| 38 | *Metagonostomum gonostomoidum* pop1 | PZ307441 | | |
| 39 | *Metagonostomum gonostomoidum* pop2 | MT247886 | MT247889 | MT247891 |
| 40 | *Metagonostomum gonostomoidum* pop3 | MT247895 | MT247899 | MT247897 |
| 41 | *Metahymena inquieta* | PV781201 | PV781206 | PX789315 |
| 42 | *Neokeronopsis asiatica* | PV667853 | PV667844 | PV667864 |
| 43 | *Notohymena gangwonensis* | MN977117 | | |
| 44 | *Oxytricha granulifera* pop1 | AF508762 | | |
| 45 | *Oxytricha granulifera* pop2 | MG836534 | | |
| 46 | *Paradiophrys zhangi* | FJ870076 | JF694077 | JF694060 |
| 47 | *Paragonostomoides xianicum* | KY451728 | MT232572 | MT240487 |
| 48 | *Paraurostyla viridis* | AF508766 | | |
| 49 | *Paraurostyla weissei* | PV777672 | PV777679 | PV777684 |
| 50 | *Paruroleptus lepisma* | AF508765 | | |
| 51 | *Pleurotricha curdsi* | PV781202 | PV781207 | PX789316 |
| 52 | *Pleurotricha lanceolata* | AF508768 | | |
| 53 | *Pseudokahliella marina* | KM222095 | KM222041 | KM222133 |
| 54 | *Pseudokeronopsis flava* | DQ227798 | DQ503579 | JQ424835 |
| 55 | *Pseudostylonychia obliquocaudata* | ON054917 | PQ013660 | |
| 56 | *Schmidingerothrix elongata* | KY085934 | MT229893 | MT229892 |
| 57 | *Schmidingerothrix salina* | FJ870085 | KM222033 | KM222121 |
| 58 | *Steinia sphagnicola* | PV781203 | PV781208 | PX789317 |
| 59 | *Sterkiella histriomuscorum* | FJ545743 | | |
| 60 | *Sterkiella nova* | AF508771 | | |
| 61 | *Stichotricha aculeata* | PX972694 | | |
| 62 | *Stichotricha koreana* | PX972693 | | |
| 63 | *Stylonychia mytilus* | AF508774 | | |
| 64 | *Stylonychia lemnae* | AF508773 | | |
| 65 | *Tetmemena* cf. *pustulata* | PQ037623 | | |
| 66 | *Tetmemena indica* | PQ037622 | | |
| 67 | *Tetmemena polymorpha* | OQ780393 | PQ013659 | |
| 68 | *Tetmemena pustulata* | AF508775 | | |
| 69 | *Thigmokeronopsis stoecki* | EU220226 | GQ246485 | JQ424844 |
| 70 | *Uroleptopsis citrina* | GU437211 | JQ424858 | JQ424850 |
| 71 | *Uroleptus gallina* | AF508779 | | |
| 72 | *Uroleptus piscis* | AF508780 | | |
| 73 | *Wallackia bujoreani* | MT247901 | MT247890 | MT247893 |
| 74 | ***Wallackia koreana* sp. nov.** | **PZ307438** | | |
